# Annulation Methods toward the Total Synthesis of Thermorubin: Construction of the AB and BCD Ring Systems

**DOI:** 10.1021/acs.joc.5c01129

**Published:** 2025-07-29

**Authors:** Zachary A. Kohanov, Andrew N. Lowell

**Affiliations:** † Department of Chemistry, 1757 Virginia Polytechnic Institute and State University (Virginia Tech), Blacksburg, Virginia 24061, United States; ‡ Center for Emerging, Zoonotic, and Arthropod-Borne Pathogens, 1757 Virginia Polytechnic Institute and State University (Virginia Tech), Blacksburg, Virginia 24061, United States; § Faculty of Health Sciences, 1757 Virginia Polytechnic Institute and State University (Virginia Tech), Blacksburg, Virginia 24061, United States

## Abstract

New antibiotics are desperately needed to fight the growing threat of antimicrobial resistance. Thermorubin, a forgotten natural product, is one such candidate, as it binds to a novel site on the ribosome and uniquely disrupts both elongation and termination during protein synthesis. In this study, we report the synthesis of the AB and BCD ring portions of thermorubin in a 10% yield over six steps and an 8.9% yield over seven steps, respectively. The key disconnection for both systems involved identification of a symmetric nucleophilic donor synthon suitable for annulation with Michael acceptors within the core tetracycle—an unusual planar aromatic system. An activated form of this symmetric intermediate enabled a formal [4 + 2] cycloaddition with a 6-carboxy pyrone as well as traditional α-β-unsaturated ketones. Beyond advancing the total synthesis of thermorubin, these strategies offer broader utility for the construction of other complex heterocycles.

## Graphical abstract

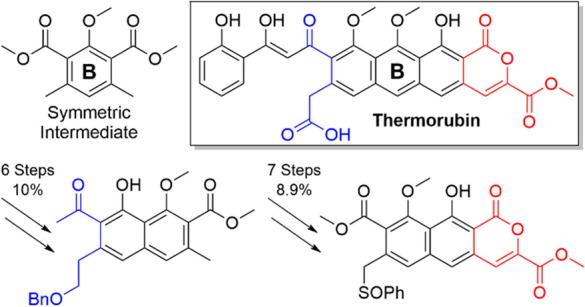

## Introduction

One of the largest problems that society faces in the 21st century is the growing threat of antimicrobial resistance. The inherent ability of bacteria to overcome antibiotics by horizontal gene transfer and mutations inexorably leads to resistance against these drugs. Even last resort drugs are susceptible to resistance genes, preventing their effective use.^3,4^ While generational improvements of established antibiotic classes continue to be important in the fight against antibiotic-resistant pathogens, it is crucial to develop new active drug scaffolds with novel mechanisms of action.

The astounding complexity of naturally produced metabolites is a critical source of antibiotics, making isolating and characterizing new scaffolds of high importance.^5^ Beyond identifying previously unknown scaffolds, new drugs can also be derived from forgotten antibiotics, molecules that have been previously reported to exhibit antibacterial properties but were not developed.^6,7^ This approach could be more efficient than de novo discovery by overcoming the time needed to collect, extract, isolate, and characterize new metabolites from biological sources, as well as avoiding the chance of rediscovery of previously known molecules.

One such forgotten antibiotic is thermorubin (**1**, Figure 1),^8^ a tetracyclic molecule^9^ that displays promising activity against pathological infections from Gram positive bacteria. It is distinguished from other biologically active molecules, such as tetracyclines and quinone-type molecules, in that most of its functionality is located along one side of the ring. Thermorubin was first isolated by Craveri^8^ and colleagues in 1964, with an initial structure^10^ being proposed in 1971 using NMR and corrected in 1980 using crystallography.^9^ Along with its unique structure, thermorubin has a unique mechanism of action, binding near the intersubunit ribosomal bridge within the bacterial ribosome.^11^ Initially thought to inhibit translation initiation,^11^ this molecule inhibits elongation and termination steps by preventing the delivery of aminoacyl-tRNA and release factor binding within the A-site.^12,41^ The high binding affinity that it has for the bound ribosomal complex as opposed to that of either individual unit also prevents the complex from disassociating, further reducing ribosomal productivity by stalling ribosome recycling.

**Figure 1:**
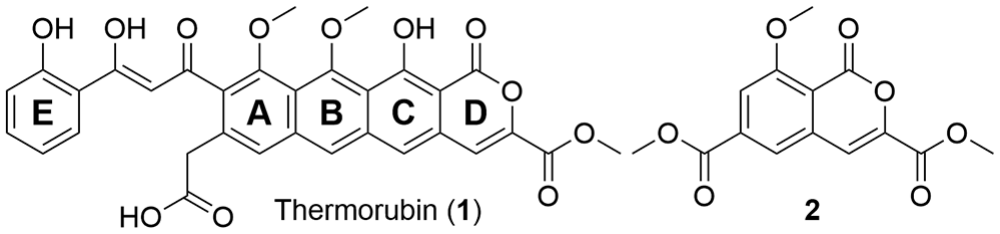
Natural product thermorubin (**1**) and previous synthetic success^15^ to the CD ring (**2**).

Our interest in thermorubin is rooted in this promise as a potential antibiotic, but direct semisynthetic derivatization^13^ was hindered by a lack of commercial availability and inconsistently reproducible growth and isolation procedures. It was, therefore, advantageous to create a synthetic route to make thermorubin and study its derivatives. Besides work^14^ focused on the initially proposed structure,^10^ the only previous attempt to synthesize thermorubin was reported by Johnson et al. in 1986.^15^ Their efforts aimed to construct the tetracyclic core of the metabolite through a variety of creative strategies, ultimately yielding an intermediate containing the CD ring system (**2**) in 6% yield over 17 steps. Inspired by this early work, we believed that annulations could not only make the aromatic core but also be used to regioselectively install functionality. After disconnection of the E ring salicylate extension (**3**, Figure 2) from the tetracyclic core (**4**), our analysis identified a masked symmetrical phthalide (**6**) within the B-ring that could act twice as a nucleophilic donor with appropriate Michael acceptors in sequential [4 + 2] cycloadditions to form the ABCD core. The core ring system (**4**) could thus be disconnected in one of two stages: either implementing annulation of the unsaturated ketone **5** with phthalide **6**, creating the AB system followed by connection of the D ring; or annulation of the phthalide **6** with the methyl ester pyrone **7**, creating the BCD system followed by a second annulation to form the A-ring.

**Figure 2:**
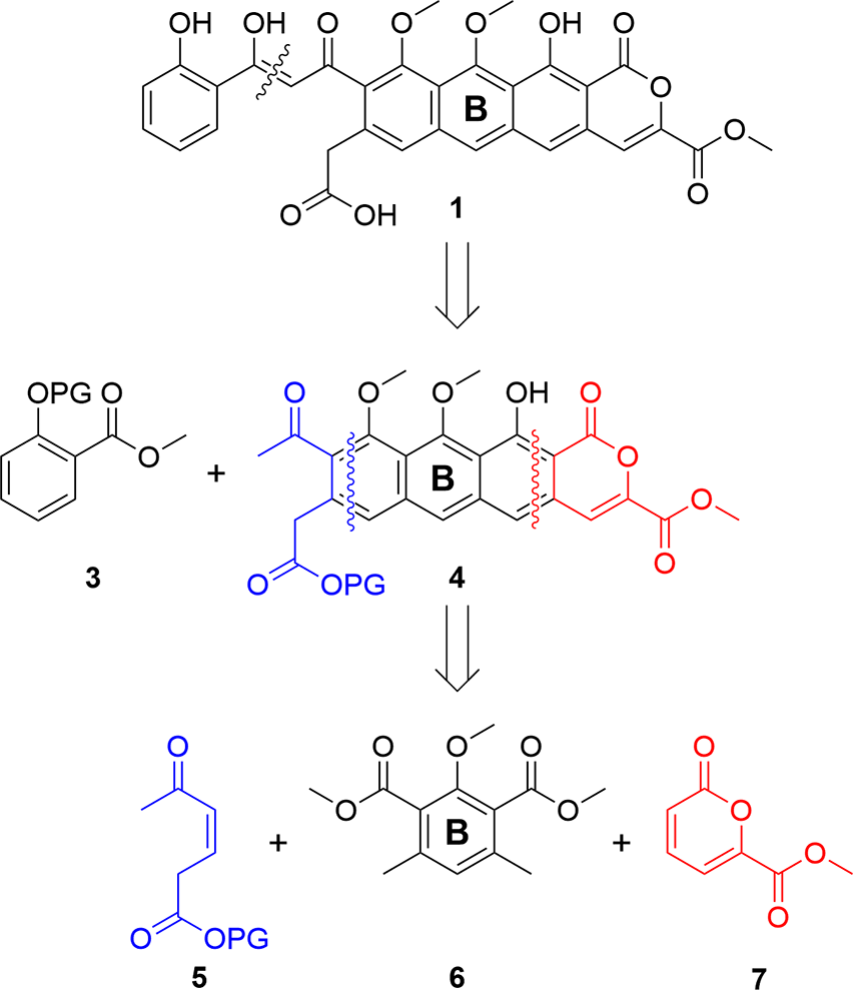
Proposed retrosynthesis of thermorubin (**1**).

## Results and Discussion

Initial examination of the literature showed the reaction of nucleophilic donors (such as **6**) with various unsaturated ketones as Michael acceptors (such as **5**) to be strongly precedented. However, while we have subsequently detailed its use,^16^ pyrones of type **7** as Michael acceptors in formal [4 + 2] cycloaddition reactions were unknown at the time and potentially complicated by regioselectivity issues. Practically, the symmetrical intermediate **6** could be used as drawn in a Staunton–Weinreb approach^17–19^ where deprotonation of a benzylic position initiates its activity as a nucleophile for reaction with **5** or **7** (or their derivatives) in a Michael addition sense, followed by a Dieckmann condensation and aromatization. Alternatively, the benzylic positions of **6** could be activated with functional groups to enhance their acidity and enable other reactions—such as a Hauser-type^20,21^ annulation—to be pursued. As all precedented Staunton–Weinreb annulation conditions act upon cyclic Michael acceptors,^22^ reaction was not attempted on the unsaturated ketone **5** or its derivatives. Additionally, all Staunton–Weinreb annulations require a leaving group on the Michael acceptor β to the activating carbonyl, meaning **7** would need to be modified to successfully reachieve aromaticity using this annulation.

Thus, testing annulation conditions utilizing 3-methoxy pyrone **8** ^17,23^ (Scheme 1) as the Michael acceptor was the initial focus. Despite precedent, the use of pyrone **8** as a Michael acceptor with isophthalate **6** ^24,25^ was unsuccessful, with both starting materials recovered. We attribute this lack of reactivity to the external ester and aromatic character of **8**, which reduced its reactivity compared to pyrones from previous studies, such as in the synthesis of toralactone.^17^

**Scheme 1:**
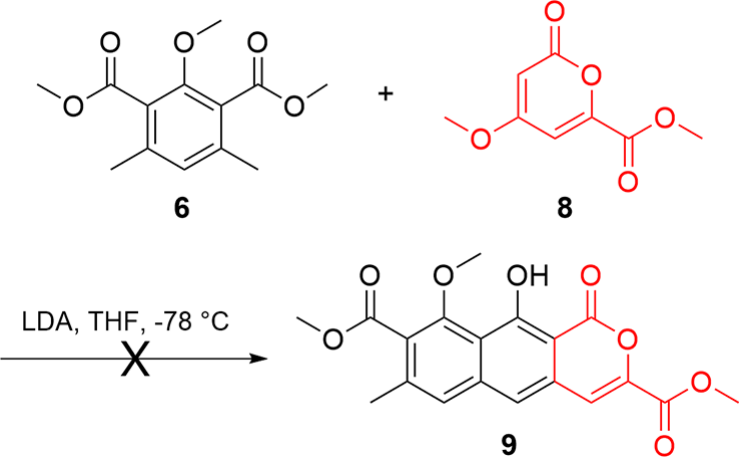
Attempted Annulation of **6** and **8** to Form **9**

This lack of reactivity in the Staunton–Weinreb-type annulation with **8** caused us to shift focus to the Hauser–Kraus annulation, where electron-withdrawing groups—such as SOPh, SO_2_Ph, CN, and halogens in the benzylic position of **6** and related isobenzofuranones—help facilitate reactivity. With the phthalide **6** already constructed, direct installation of the sulfoxide functionality (**12**, Scheme 2) as an electron-withdrawing group was attempted using base and methylbenzenesulfinate,^26^ but these conditions were ineffective. Similarly, treatment of **6** with base and diphenyldisulfide^27,28^ failed to produce **11** directly. However, a longer sequence^29^ eventually proved successful. Radical bromination of symmetrical isophthalate **6** provided a difficult-to-purify mixture of the desired monobromide (**10**) and polybrominated byproducts. This mixture was carried forward through an S_N_2 displacement with thiophenol to give another mixture containing the desired thioether **11**. After oxidation of thioether **11** to sulfoxide **12** with sodium periodate, the mixture was successfully separated, yielding sulfoxide **12** in a 32% yield over three steps.

**Scheme 2:**
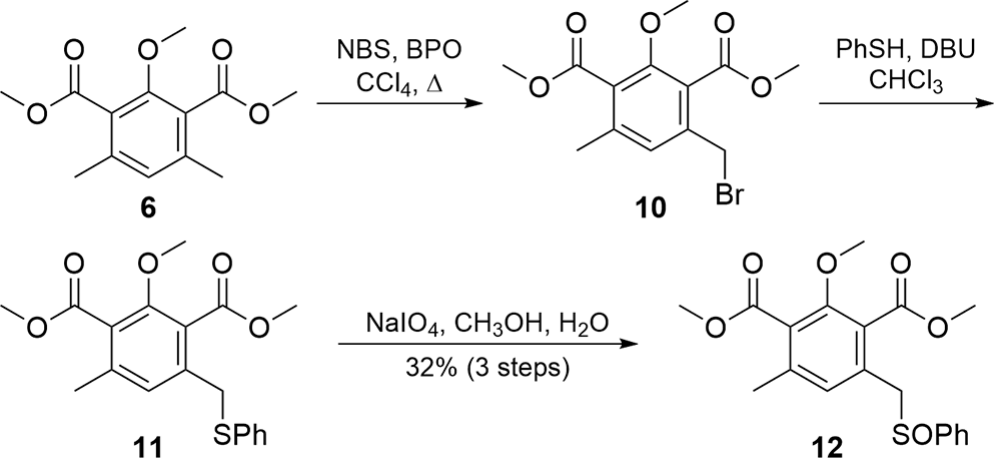
Synthesis of Monosulfoxide **12**

With a suitably activated, monofunctionalized nucleophilic donor in hand (**12**), our attention turned to the unsaturated ketone synthon and creation of the AB ring. If a dicarbonyl-containing system were to be used, isomerization of the internal bond would occur readily^30^ (**5**_**a**_ and **5**_**b**_, Figure 3). This enol-driven tautomerization would diminish yields because of annulation occurring at the α-β-unsaturated ester tautomer in addition to the desired α-β-unsaturated ketone location. To overcome this potential problem, we targeted a protected alcohol (**13**) in lieu of the ester.

**Figure 3:**
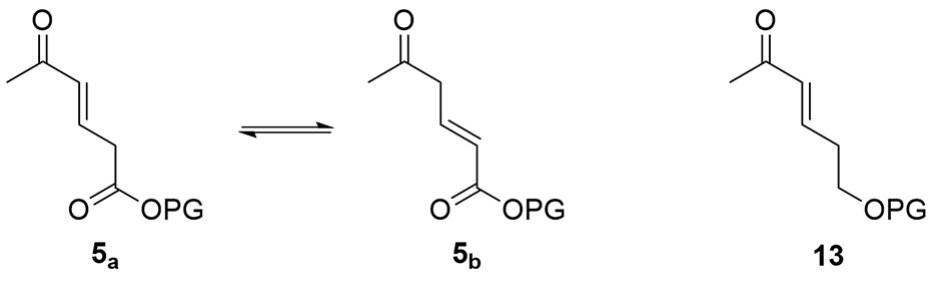
Tautomerization of unsaturated ketone **5** and its less oxidized partner **13**, a regioselective alternative.

General reaction procedures based on the seminal work of Hauser and colleagues^31^ (Scheme 3) called for a slight excess of the Michael acceptor to be added slowly to a cold solution of the nucleophilic donor premixed with base. When applied to this system, the slow addition of **13** to **12** resulted in the annulated product **14** in 39% yield. Methylation of the phenol of **14** proceeded in 88% yield to give **15**.

**Scheme 3:**
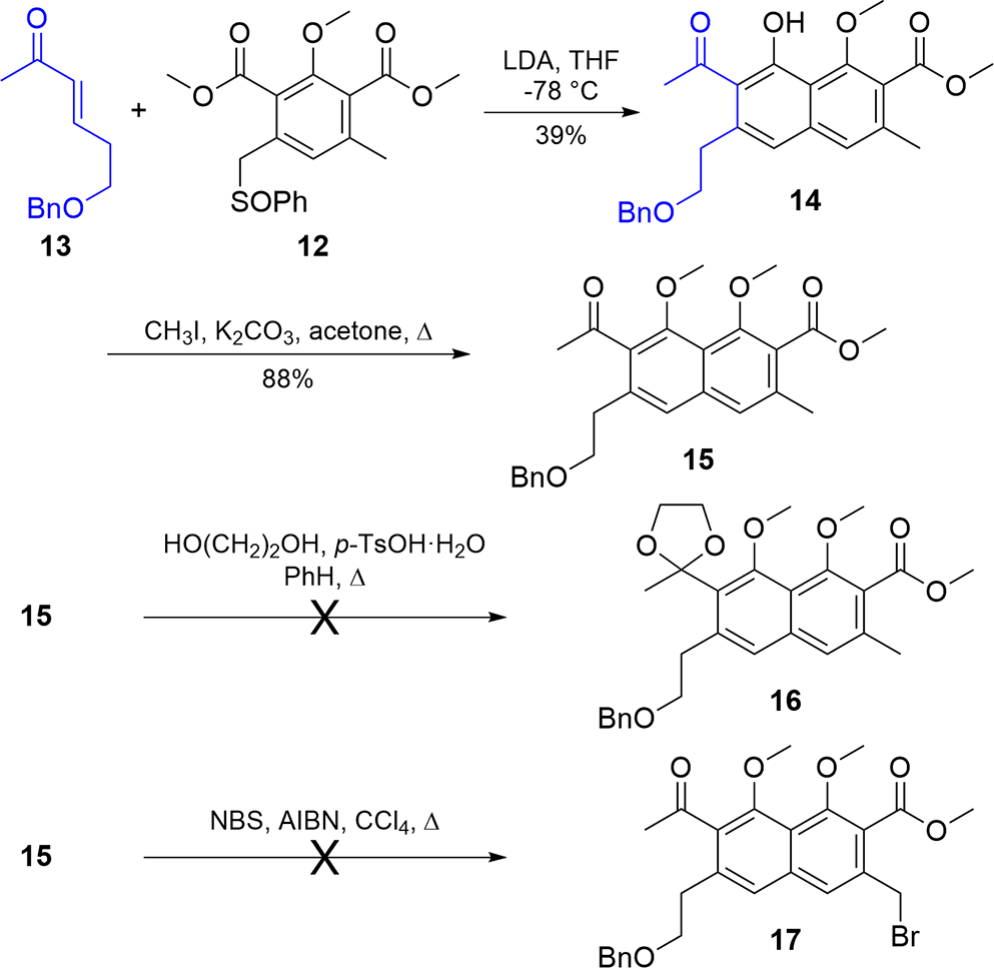
Annulation of **13** and **12** to Form the AB Ring (**14**) and Progression Attempts

Despite this initial success in producing the AB ring of thermorubin, bicycle (**15**) could not be further propagated. When attempting to protect the external ketone as the dioxolane (**16**)—a conversion needed to temper the acidity of the position α to the ketone and enable subsequent annulations at the benzylic site—the protected intermediate could only be generated in a 1:8 ratio relative to the starting material, likely due to steric interference from the neighboring groups. An alternative strategy sought to brominate the benzylic methyl group (**17**), but due to a lack of directing groups and the presence of an additional benzylic carbon, bromination was not regioselective. Instead, an inseparable mixture of differentially brominated materials was generated.

Because the completed AB ring system could not be advanced toward the thermorubin core, annulation of a phthalide, such as **12**, with the methyl ester pyrone moiety (**7**)^16^ was the next logical approach. If successful, this would allow for direct annulation of the intact D ring pyrone to the symmetric B-ring phthalide, forming the BCD system. As Hauser–Kraus-type reactions using fully unsaturated^32^ pyrones as regioselective Michael acceptors had not been reported, we created chemistry that enabled this annulation,^16^ using a model system to demonstrate the utility of a wide variety of 6-carboxypyrones, such as **7**, in annulation reactions.

Buoyed by this success, we turned our attention back toward a molecule suitable for progression to thermorubin (Figure 4). Viable B-ring candidates for reaction with **7**, an intermediate whose synthesis and reactivity were established in our earlier work,^16^ were previously constructed monosulfoxide **12** or its symmetric counterpart, disulfoxide **18**. Monosulfoxide **12** would not suffer from potential overreaction^27^ but would require subsequent installation of another activating sulfoxide to complete formation of the A-ring. Disulfoxide **18** might suffer from overannulation but would avoid the late-stage sulfoxide installation that derailed the advancement of **15**.

**Figure 4:**
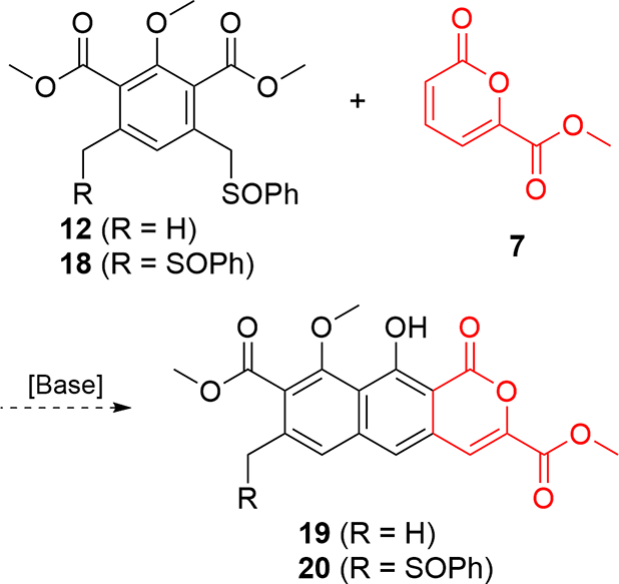
Potential activated phthalides for reaction with pyrone **7**.

Initial efforts focused on using monosulfoxide **12** (Scheme 4) with pyrone **7** under the previously established conditions,^16^ furnishing **19** in a 33% yield. The annulated material was protected as the silyl ether (**21**). Several attempts were made to activate the C7 benzylic position of **21** to carry this material forward through A-ring formation. Conditions identical to those applied to the symmetrical phthalide resulted in nonregioselective halogenation, yielding electrophilic aromatic substitution products^33^ that were inseparable from **22**. Direct sulfonylation^26^ (**23**) and sulfinylation^27,28^ (**24**) likewise failed although carbanion generation from the addition of LDA was apparent by a bright color change, suggesting methyl benzenesulfinate and diphenyldisulfide are insufficient electrophiles for this material.

**Scheme 4:**
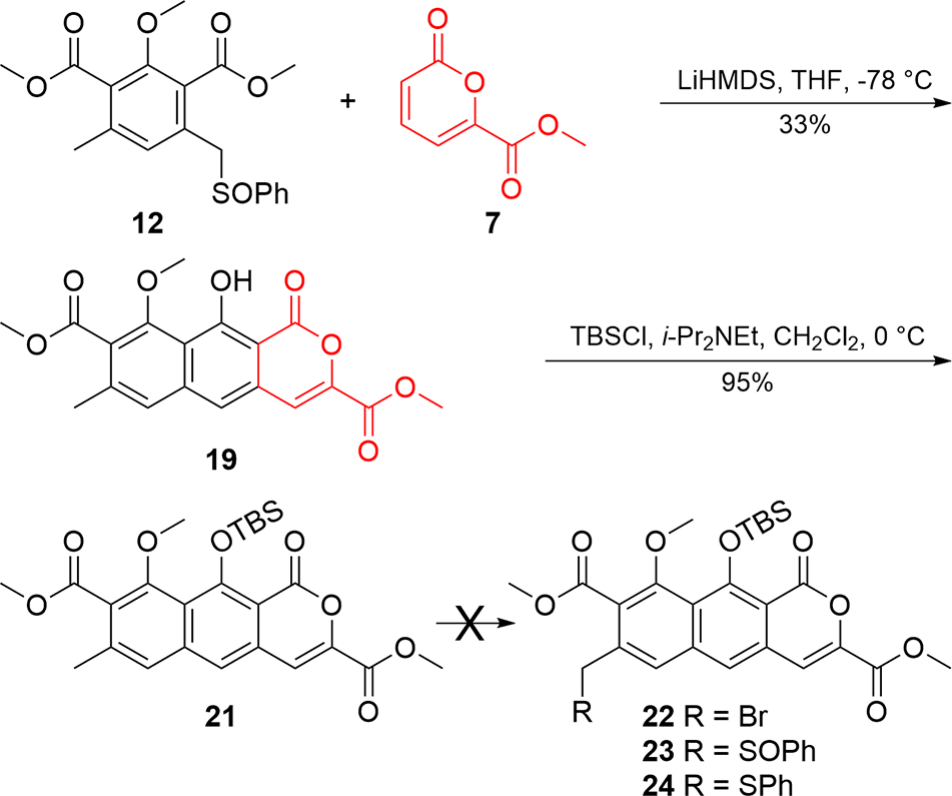
Annulation Using Pyrone **7** and Monosulfoxide **12** to Synthesize BCD Tricycle **21** and Attempted Activation

With the activation and advancement of **21** not possible, our focus shifted to the synthesis and use of a disulfoxide B-ring (**18**). Modification of the previously generated monosulfoxide material **12** (Scheme 5) was initially attempted but resulted in nonregioselective bromination rather than **25**. Symmetric dibromination of the initial phthalide **6** was also attempted, but use of two or more equivalents of the brominating agent resulted predominantly in dibromination at a single benzylic position (**26**). The differentially brominated products were inseparable, and this approach was abandoned.

**Scheme 5:**
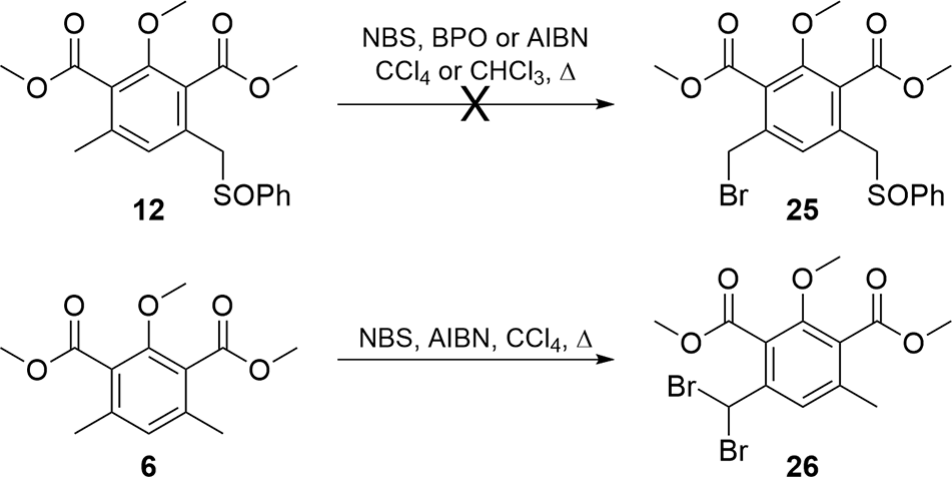
Attempted Synthetic Routes to Disulfoxide **18** from Available Precursors

To circumvent this problem, we sought to install functional handles on the diketone (Scheme 6) prior to cyclization, which could then be converted to the disulfoxide after formation of the phenol. Initial work produced **30** via 1,5-dicholoro acetylacetone,^34,35^ but dibromination^36,37^ of ethyl diacetoacetate (**27**) to give **28** followed by decarboxylation produced a dihalogen (**29**) more reliably. Subsequent displacement of the bromides on **29** with thiophenol resulted in dithiophenylether acetylacetone (**30**) in the best overall yield.

**Scheme 6:**
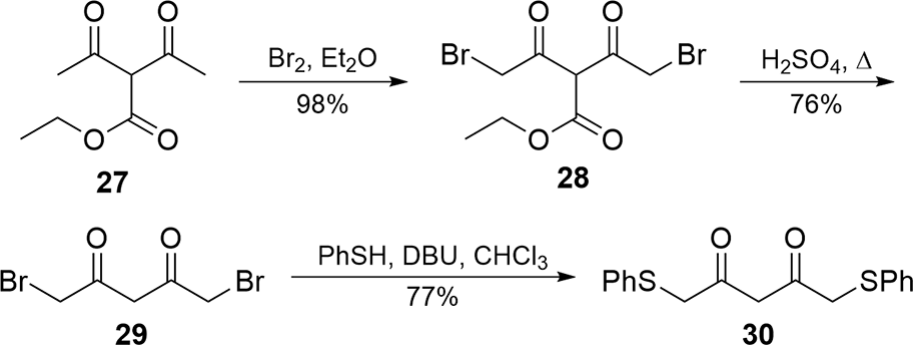
Formation of the Difunctionalized Acetoacetone **30**

With an efficient route to disubstituted acetoacetone **30** in hand, our attention turned to cyclization of this material to construct **18** as a diactivated B-ring. The original precedent for synthesis of phthalates specifically used unsubstituted acetylacetone for cyclization with oxoglutarate because while more sterically hindered acetylacetones would cyclize, they did so in poorer yields.^24^ This decrease in yield was observed in the reaction of **31** with **30** (Table 1, Entry 1), compared to a yield of 86% when the unsubstituted acetylacetone was used (see S7, Scheme S1). Increasing the equivalencies of base (entry 2), temperature (entry 3), or reaction time (entries 4 and 5) did not increase product formation. Decreasing the initial concentration of water by adding solid sodium hydroxide (entries 6 and 7) promoted formation of the product compared to corresponding aqueous reactions (entries 4 and 5). However, extended reflux (entry 8) resulted in no product. In initially anhydrous conditions using sodium to generate methoxide, product formation was the highest with a slight excess of base (entry 10, 56% yield based on the recovered starting material) being optimal compared to an equimolar amount (entry 9). Other reaction conditions were attempted using different solvents and bases (entries 11–14) but, with the exception of triethyl amine, were not as high-yielding as the sodium-mediated or sodium hydroxide-mediated reactions.

**Table 1:**
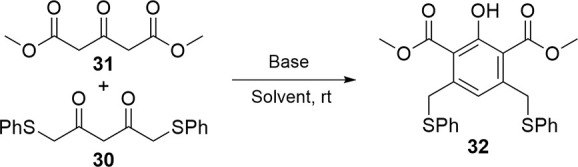
Optimization of Cyclization Conditions to Form **33**

entry	base	equiv of base	solvent	time (h)	% yield
1	NaOH_(aq)_	0.5	CH_3_OH	48	40
2	NaOH_(aq)_	1.0^a^	CH_3_OH	48	32
3	NaOH_(aq)_	1.0	CH_3_OH	25^b^	33
4	NaOH_(aq)_	0.1	CH_3_OH	312	36
5	NaOH_(aq)_	1.0	CH_3_OH	312	13
6	NaOH_(S)_	0.1	CH_3_OH	48	45
7	NaOH_(S)_	1.0^a^	CH_3_OH	48	26
8	NaOH_(S)_	1.0^a^	CH_3_OH	48^c^	0
9	Na	1.0	CH_3_OH	120	35
10	Na	1.1	CH_3_OH	80	40^d^
11	NaH	1.1	THF	60	10
12	TEA	3.0	CH_3_CN	72	40
13	DBU	1.5	CH_3_CN	12	26
14	TMP	1.5	CH_3_CN	12	25

a Additional 0.5 equiv of base added halfway through the reaction.

b Then refluxed for 1 h.

c Then refluxed for 24 h.

d 56% yield based on recovered starting material.

Synthesis of diphenylsulfoxide **18** (Scheme 7) proceeded using reaction conditions established with earlier systems. Phenol **32** was methylated under basic conditions,^25^ giving **33** in a 99% yield. Oxidation of the thioether moieties of **33** proceeded in a high yield with 2.5 equivalencies of sodium periodate to obtain the symmetric disulfoxide **18**. Notably, when fewer equivalents of oxidant were used, the mono sulfoxide (**34**) could also be isolated. While annulation with **46** was not pursued, it could be produced intentionally from **33** in up to a 70% yield.

**Scheme 7:**
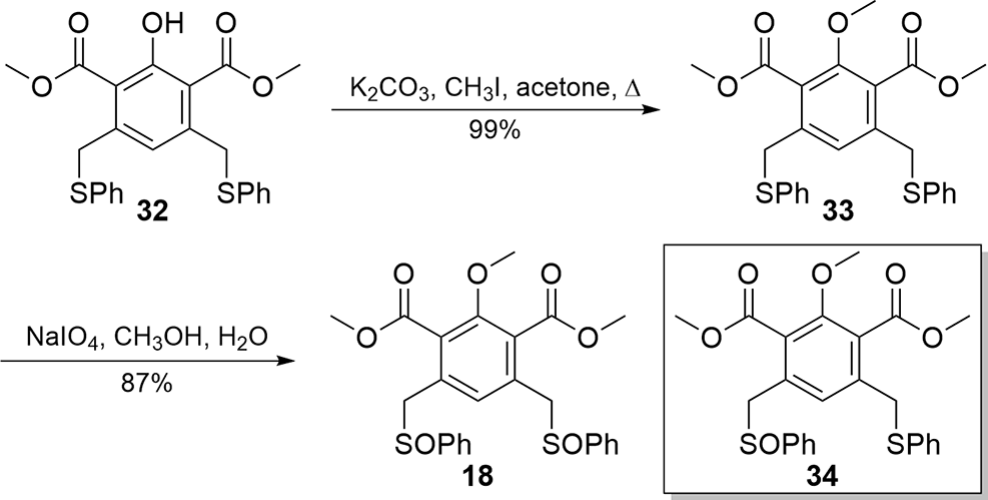
Formation of the Disulfoxide Intermediate **18**

As per our earlier findings,^16^ it was clear that the [4 + 2] cycloaddition of **18** (Table 2) with pyrone **7** would require optimization. Application of the previously optimized conditions^16^ only produced **20** in a 19% yield (entry 1). Using less equivalencies of base with a slight excess of pyrone resulted in lower yields (entries 2–3), which tracks with the proposed mechanism.^16^ Extended reaction times (entry 4) did not improve yields, but increasing the amount of pyrone **7** appeared to help product formation slightly (entries 5 and 6). The best results were obtained (entry 7) when 4 equiv of base and 2.4 equiv of pyrone were used, giving a 32% yield. Four equivalents of base are likely required because the other methylene is a fourth basic center that must be deprotonated during the reaction. Despite the additional equivalency of base and reports of multiple additions with other systems,^27^ no diannulated material was observed. We believe this is because the disulfoxide benzylic positions in this system are conjugated, whereas in previous studies, the two sulfoxides were borne on separate aromatic systems.^27^

**Table 2:**
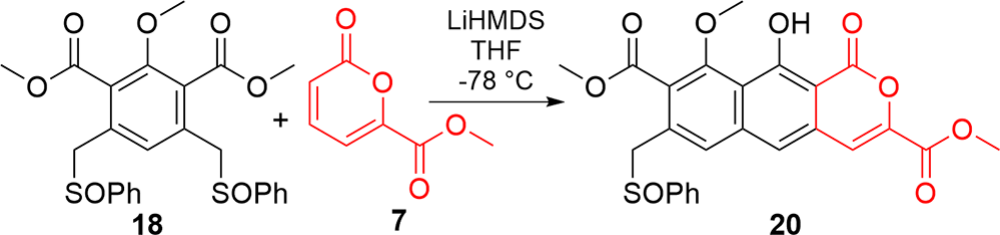
Optimization of Disulfoxide (**18**) Annulation with Methyl Ester Pyrone (**7**)

entry	base equiv	pyrone equiv	time	% yield
1	3.0	2.4	1	19
2	1.5	1.1	1	0
3	2.5	1.1	1	18
4	3.0	1.1	12	12
5	3.0	2.0	1	23
6	3.1	1.8	1	17
7	4.0	2.4	12	32

Toward installing the A-ring on **20** (Scheme 8), we investigated protecting groups for the central phenol that would enable a subsequent annulation. Initially, *para*-methoxy benzyl (PMB) was selected as a sufficiently robust, orthogonal protecting group, but surprisingly, treatment of **20** with PMBCl resulted in electrophilic aromatic substitution (**35**) rather than the intended alkylation of the phenol, presumably due to steric hindrance disfavoring the phenolate as a nucleophile. Based on our earlier success with **19**, we instead silylated the phenol (**36**), albeit in a lower yield, presumably because of the same gearing effect from the phenylsulfoxide hindering reaction at the phenol. While successful at masking the phenol, reaction of **36** with **13** did not produce an isolable amount of **37**, despite repeated attempts. However, masses consistent with both the desired product **37** and its desilylated counterpart **38** could be detected in the reaction mixture. While these results indicated that protected derivatives of **20** are suitable for annulation reactions, they emphasize the critical role of protecting groups. In this case, these densely functionalized planar aromatics were held back by solubility difficulties compounded by challenging separations of similar byproducts. Thus, future work will focus on identifying a protecting group that withstands annulation and enables efficient purification.

**Scheme 8:**
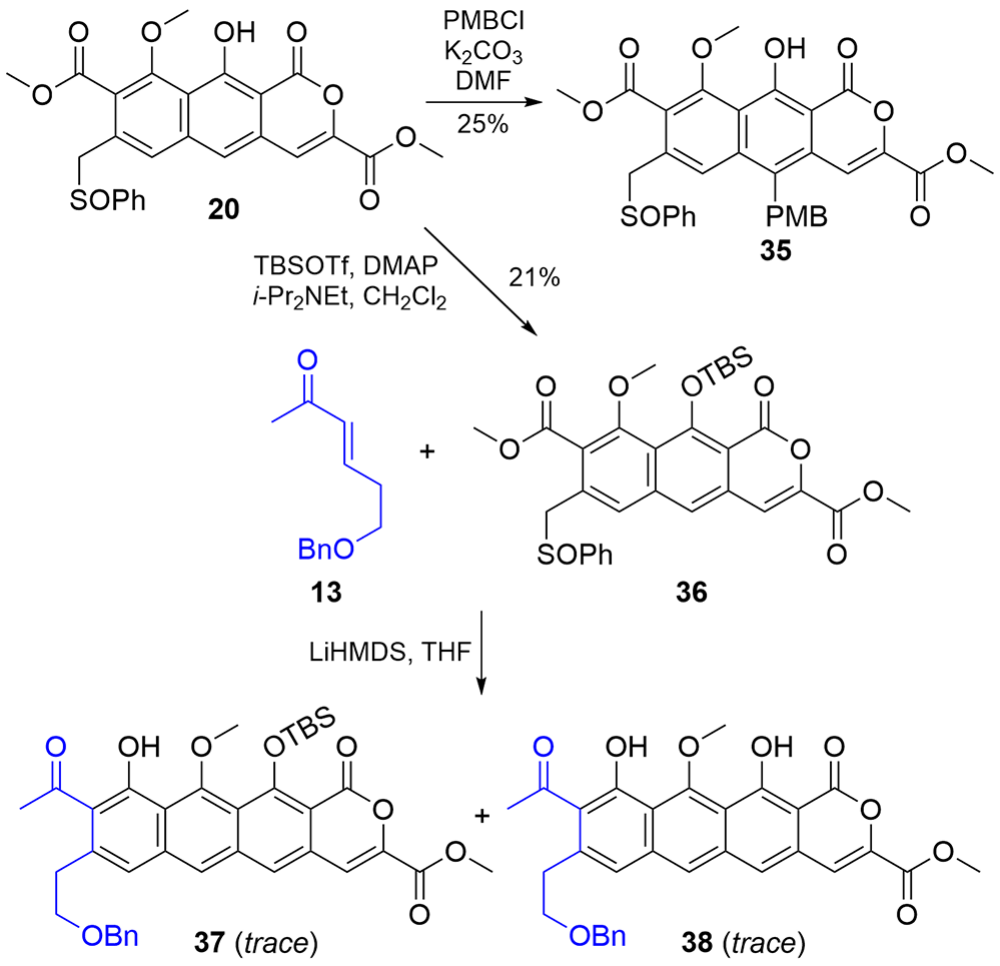
Protection and A-Ring Annulation Attempt

## Conclusions

This work expands the available chemistry for synthesizing highly functionalized, planar aromatic molecules by completing annulations toward the total synthesis of thermorubin. The optimization of a Michael acceptor pyrone—6-methoxy-2-pyrone carboxylate (**7**)—in a Hauser-type annulation was the key step that enabled formation of the BCD ring of thermorubin. The overall process for the BCD ring (**20**) proceeded in an 8.9% yield over seven linear steps (ten total). Suitable protection conditions for this intermediate, such as methyoxymethylene, that may be sterically small enough to favor attachment at the phenol rather than the EAS yet sufficiently robust to withstand the basic conditions, would support annulation of the A-ring and completion of the thermorubin core ring system. This work also successfully produced the AB ring of thermorubin (**14**) in a 10% yield over six linear steps (ten total). Although this fragment could not be converted into an activated naphthalene suitable for annulation with pyrone **7**, an alternative approach that avoids symmetric intermediates of type **6** and instead directly yields a suitably functionalized naphthalene in the vein of **17** may permit successful coupling with **7**. Thus, while conditions for assembling the complete ABCD core and attaching the E ring remain to be optimized, this study demonstrates how a novel, symmetric synthon strategy—leveraging annulation with an intact pyrone D ring—enables construction of the majority of the thermorubin core.

## Experimental Section

### General

Chemical reagents and solvents were purchased from EMD Millipore, Oakwood Chemical, Sigma-Aldrich, Beantown Chemical, Acros, and Thermo Fisher Scientific. Unless otherwise specified, all nonaqueous reactions were carried out under an atmosphere of dry nitrogen in dried glassware. Commercially available starting materials and reagents were used as received or purified prior to use, if necessary. Anhydrous^38^ THF was obtained commercially or from a solvent purification system.^39^ Diisopropylethylamine was distilled from calcium hydride. *n*-BuLi was titrated using 3,5-di-*tert*-butyl-4-hydroxytoluene in THF with fluorene as an indicator. Analytical thin-layer chromatography was performed using Supelco 0.25 mm silica gel 60 F_254_ plates. Visualization was accomplished by irradiation with a 254 nm UV lamp or by staining with a basified aqueous solution of potassium permanganate. Chromatography was performed using a forced flow^40^ of the indicated solvent system on SiliCycle SiliaFlash P60 silica gel or with a Biotage Selekt automated flash chromatography system using prepacked commercial columns. Deionized water was obtained from a house deionized water system.

^1^H NMR spectra were recorded on a Bruker Avance II 500 MHz spectrometer, an Agilent U4-DD2 400 MHz spectrometer, a Nanalysis 100 MHz spectrometer, or a Bruker Avance III 600 MHz spectrometer. Chemical shifts are reported in parts per million from tetramethylsilane (0 ppm) using the solvent resonance as an internal standard (CDCl_3_ = 7.26 ppm, CD_3_OD = 3.31 ppm). Data are reported as follows: chemical shift, multiplicity (s, singlet; d, doublet; t, triplet; q, quartet; p, pentet; m, multiplet; br, broad), coupling constant, and number of protons. Proton decoupled ^13^C NMR spectra were recorded on a Bruker Avance II 500 MHz (126 MHz) spectrometer, an Agilent U4-DD2 400 MHz (101 MHz) spectrometer, or a Bruker Avance III 600 MHz (151 MHz) spectrometer. Chemical shifts are reported in parts per million from tetramethylsilane (0 ppm) using the solvent resonance as an internal standard (CDCl_3_ 77.16 ppm, CD_3_OD 49.0 ppm). High-resolution mass spectra were obtained on an Agilent Technologies 6220 TOF LC/MS or a Waters Synapt Q-TOF G2 in the Department of Chemistry and the VT-Mass Spectrometry Incubator at the Virginia Polytechnic Institute and State University.

#### Dimethyl 2-methoxy-4-methyl-6-((phenylsulfinyl)­methyl)­isophthalate (**12**)

**Caution!** Carbon tetrachloride is highly toxic and should be handled exclusively in a fume cabinet to avoid vapor exposure. To a flame-dried round-bottomed flask was added **6** (10.3 g, 40.7 mmol), followed by NBS (7.3 g, 40.7 mmol) and benzoyl peroxide (197 mg, 0.813 mmol). The vessel was purged with nitrogen, and CCl_4_ (90 mL) was added. The stirring mixture was heated to reflux (oil bath) for 2 h. After cooling, the reaction mixture was filtered (filter paper, CCl_4_), and the filtrate was concentrated to yield **10** as an orange oil. This residue was carried onto the next step without further purification.

Impure **10** was dissolved in CHCl_3_ (100 mL), after which DBU (6.1 mL, 41 mmol) and thiophenol (4.2 mL, 41 mmol) were added. After the mixture was stirred for 24 h at rt, additional DBU (1.5 mL, 10 mmol) was added, and the mixture was stirred for an additional 24 h. A solution of aqueous NaOH (75 mL, 0.67 M) was added to the mixture, and the layers were separated. The aqueous layer was washed with Et_2_O (2 × 100 mL), and the combined organic layers were washed with aqueous NaOH (75 mL, 0.33 M), HCl (75 mL, 3 M), water (75 mL), and brine (75 mL). The organic layer was dried (Na_2_SO_4_) and concentrated under reduced pressure to yield **11** as a dark-orange oil, which was carried onto the next step without further purification.

Impure **11** was dissolved in methanol (107 mL) and water (16 mL). To this stirring solution was added NaIO_4_ (8.7 g, 41 mmol), and the mixture was stirred overnight. The mixture was filtered (filter paper), and the precipitate was washed with methanol. The filtrate was concentrated and suspended in CH_2_Cl_2_ (100 mL). The mixture was filtered (Celite, CH_2_Cl_2_), and the filtrate was concentrated. The residue was purified using automated flash chromatography (SiO_2_, 38%–45% EtOAc:hexanes) to yield sulfoxide **12** (4.8 g, 32%) as an off-white to yellow amorphous solid: ^1^H NMR (400 MHz, CDCl_3_) δ 7.53–7.42 (m, 5H), 6.69 (s, 1H), 4.16 (d, *J* = 12.7 Hz, 1H), 4.08 (d, *J* = 12.9 Hz, 1H), 3.91 (s, 3H), 3.87 (s, 3H), 3.78 (s, 3H), 2.22 (s, 3H); ^13^C­{^1^H} NMR (126 MHz, CDCl_3_) δ 167.6, 167.0, 156.1, 143.3, 139.3, 131.6, 131.4, 129.21, 129.20, 129.17, 125.3, 124.3, 63.7, 62.0, 52.7, 52.6, 19.4.; HRMS (ESI) *m*/*z*: [M + H]^+^ calcd for C_19_H_21_O_6_S 377.1059; found 377.1043.

#### Methyl 7-acetyl-6-(2-(benzyloxy)­ethyl)-8-hydroxy-1-methoxy-3-methyl-2-naphthoate (**14**)

To a flask containing THF (1 mL) was added *i*Pr_2_NH (0.180 mL, 1.28 mmol). The solution was cooled in a water/ice bath, and *n*BuLi (0.580 mL, 2.02 M, 1.17 mmol) was added dropwise. The stirring mixture was warmed to rt over 15 min and then cooled to −78 °C using a dry ice/acetone bath. After the mixture was stirred cold for 15 min, **12** (0.200 g, 0.531 mmol) dissolved in THF (1.75 mL) was added dropwise. After the mixture was stirred for 15 min, **13** (0.261 g, 1.28 mmol) dissolved in THF (1.75 mL) was added dropwise. The mixture was stirred cold for 60 min and then warmed to rt with stirring over 120 min. The mixture was quenched by the addition of aqueous HCl (3 mL, 1 M) and concentrated to remove THF. The concentrated mixture was extracted with CHCl_3_ (3 × 4 mL). The combined organic layers were dried (Na_2_SO_4_) and concentrated. The residue was purified by automated flash chromatography (SiO_2_, 2–10% acetone:CHCl_3_) to yield **14** (0.088 g, 39%) as a red amorphous solid. ^1^H NMR (400 MHz, CDCl_3_) δ 9.76 (s, 1H), 7.40–7.28 (m, 6H), 7.12 (s, 1H), 4.51 (s, 2H), 4.01 (s, 3H), 3.99 (s, 3H), 3.70 (t, *J* = 6.7 Hz, 2H), 3.01 (t, *J* = 6.8 Hz, 2H), 2.58 (s, 3H), 2.42 (s, 3H). ^13^C­{^1^H} NMR (101 MHz, CDCl_3_) δ 205.3, 168.1, 154.5, 152.3, 138.4, 137.1, 136.7, 134.8, 130.2, 128.4, 127.8, 127.7, 124.9, 123.7, 119.8, 113.6, 73.0, 70.8, 64.5, 52.7, 33.9, 32.6, 20.0; ESI calculated for C_25_H_27_O_6_ 423.1802; found 423.1788.

#### Methyl 7-acetyl-6-(2-(benzyloxy)­ethyl)-1,8-dimethoxy-3-methyl-2-naphthoate (**15**)

To a flame-dried, nitrogen-purged flask charged with K_2_CO_3_ (0.039 g, 0.28 mmol) was added **14** (0.098 g, 0.23 mmol) dissolved in acetone (6 mL). CH_3_I (0.029 mL, 0.46 mmol) was added, and the mixture was heated to reflux (oil bath) for 20 h. After cooling, the mixture was filtered (filter paper, CH_2_Cl_2_) and the filtrate was concentrated. The residue was dissolved in CH_2_Cl_2_ (6 mL), washed with water (2 × 3 mL), dried (Na_2_SO_4_), and concentrated. The residue was purified using automated flash chromatography (SiO_2_, 0–1% CH_3_OH:CHCl_3_) to yield **15** (0.088 g, 88%) as a red-orange amorphous solid: ^1^H NMR (400 MHz, CDCl_3_) δ 7.41 (s, 1H), 7.36 (d, *J* = 1.1 Hz, 1H), 7.34–7.24 (m, 5H), 4.51 (s, 2H), 3.99 (s, 3H), 3.92 (s, 3H), 3.80 (s, 3H), 3.71 (t, *J* = 7.0 Hz, 2H), 2.95 (t, *J* = 7.0 Hz, 2H), 2.56 (s, 3H), 2.41 (d, *J* = 1.0 Hz, 3H); ^13^C­{^1^H} NMR (101 MHz, CDCl_3_) δ 205.9, 168.9, 153.1, 152.7, 138.3, 137.5, 135.0, 134.7, 134.0, 128.5, 127.9, 127.8, 127.5, 125.2, 124.7, 118.6, 73.1, 70.5, 64.23, 64.19, 52.5, 33.3, 33.0, 19.6; ESI calculated for C_26_H_28_NaO_6_ 459.1784: found, 459.1784.

#### Dimethyl 10-hydroxy-9-methoxy-7-methyl-1-oxo-1*H*-benzo­[*g*]­isochromene-3,8-dicarboxylate (**19**)

To a flask containing THF (2 mL) was added HMDS (2.42 mL, 11.6 mmol), and the mixture was cooled to 0 °C using a water/ice bath. Once cooled, *n*BuLi (5.2 mL, 2.1 M) was added slowly. After addition, the mixture was warmed to room temperature, stirred for 15 min, and cooled to −78 °C using a dry ice/acetone bath. Once the mixture was cooled, pyrone **7** (1.36 g, 8.75 mmol) was dissolved in THF (45 mL) and was added slowly. After stirring for 15 min, sulfoxide **12** (1.40 g, 3.64 mmol) dissolved in THF (16 mL) was slowly added dropwise. After stirring cold for 60 min, the cold bath was removed and the reaction was left to stir for an additional 60 min. The reaction was quenched by the addition of aqueous 10% HCl (24 mL), and residual THF was removed under reduced pressure. The resulting mixture was diluted with CHCl_3_ (30 mL), and the layers were separated. The aqueous layer was extracted with CHCl_3_ (2 × 30 mL). The combined organic layers were dried (Na_2_SO_4_) and concentrated. Purification using automated flash chromatography (SiO_2_, 1 to 10% EtOAc:CHCl_3_) yielded **19** (0.46 g, 33%) as a yellow amorphous solid: ^1^H NMR (400 MHz, CDCl_3_) δ 12.89 (s, 1H), 7.51 (s, 1H), 7.44 (s, 1H), 7.34 (s, 1H), 3.992 (s, 3H), 3.990 (s, 3H), 3.98 (s, 3H), 2.46 (s, 3H); ^13^C­{^1^H} NMR (100 MHz, CDCl_3_) 168.0, 166.4, 163.1, 160.6, 156.1, 141.8, 140.3, 139.1, 130.1, 128.7, 124.9, 117.01, 116.95, 114.1, 101.2, 64.5, 53.1, 52.6, 19.9; HRMS (ESI) *m*/*z*: [M + H]^+^ calcd for C_19_H_17_O_8_ 373.0923, found 373.0907.

#### Dimethyl 10-((*tert*-butyldimethylsilyl)­oxy)-9-methoxy-7-methyl-1-oxo-1*H*-benzo­[*g*]­isochromene-3,8-dicarboxylate (**21**)

To a flask containing **19** (0.024 g, 0.064 mmol) was added CH_2_Cl_2_ (1.5 mL). Once fully dissolved, the mixture was cooled in an ice/water bath, and DIPEA (0.170 mL, 0.999 mmol) was added dropwise. After the mixture was stirred for 1 min, TBSCl (0.0970 g, 0.642 mmol) was added. After being stirred for 5 min, the reaction was warmed to room temperature and stirred overnight. The reaction was quenched by the addition of a saturated aqueous solution of NaHCO_3_ (2 mL), and the layers were separated. The aqueous layer was washed with CH_2_Cl_2_ (3 × 2.5 mL), and the combined organic layers were dried using Na_2_SO_4_ and concentrated. The residue was purified using automated flash chromatography (SiO_2_, 20% to 50% EtOAc:CHCl_3_) to yield **21** (0.029 g, 95%) as an amorphous yellow solid. ^1^H NMR (400 MHz, CDCl_3_) δ 7.47 (s, 1H), 7.42 (s, 1H), 7.40 (s, 1H), 3.98 (s, 3H), 3.95 (s, 3H), 3.81 (s, 3H), 2.43 (s, 3H), 1.11 (s, 9H), −0.01 (s, 6H); ^13^C­{^1^H} NMR (101 MHz, CDCl_3_) δ 168.0, 161.2, 158.6, 157.9, 155.8, 142.4, 139.4, 137.6, 132.2, 128.0, 124.2, 121.1, 119.6, 112.5, 110.0, 63.6, 52.8, 52.4, 26.2, 19.6, 18.5, −4.4; HRMS (ESI) *m*/*z*: [M + H]^+^ calcd for C_25_H_31_O_8_Si 487.1788, found 487.1783.

#### Ethyl 4-bromo-2-(2-bromoacetyl)-3-oxobutanoate (**28**)

The following reaction was carried out in an analogous manner to the published procedure.^37^ To a 2-necked round-bottomed flask equipped with an addition funnel and an outlet to a water bath was added **27** (9.99 g, 58.0 mmol) and Et_2_O (40 mL). The mixture was cooled in a water/ice bath, and bromine (6.00 mL, 117 mmol) was added dropwise over 2 h. The mixture was warmed to room temperature and stirred overnight. After pouring onto ice (50 g), the mixture was diluted with Et_2_O (50 mL). The layers were separated, and the organic layer was washed with water (2 × 50 mL) and brine (25 mL), dried (Na_2_SO_4_), and concentrated to give **28** (18.7 g, 98%) as an orange oil that solidified upon cooling to an orange-brown amorphous solid. ^1^H NMR (400 MHz, CDCl_3_) δ 4.39 (s, 4H), 4.37 (br s, 1H), 4.35 (q, *J* = 7.2 Hz, 2H), 1.40 (t, *J* = 7.2 Hz, 3H); ^13^C­{^1^H} NMR (101 MHz, CDCl_3_) δ 191.6, 164.9, 106.5, 61.9, 30.0, 14.0; HRMS (ESI) *m*/*z*: [M + Na]^+^ calcd for C_8_H_10_Br_2_NaO_4_ 350.8844; found 350.8834.

#### 1,5-Dibromopentane-2,4-dione (**29**)

The following reaction was carried out in an analogous manner to the published procedure.^37^ Compound **28** (28.7 g, 87.0 mmol) was dissolved in conc. H_2_SO_4_ (40 mL). The mixture was slowly heated (oil bath) with appropriate venting to an internal temperature of 85 °C, and this temperature was maintained for 15 min. The reaction mixture was cooled to room temperature, after which it was poured onto ice-cold water (850 mL). After the ice had melted, the aqueous mixture was extracted with Et_2_O (3 × 250 mL). The combined organic layers were washed with brine (250 mL), dried (Na_2_SO_4_), and concentrated under a nitrogen stream. The residue was purified using automated flash chromatography (SiO_2_, 8–10% EtOAc:Hex) to yield **29** (17.0 g, 76%) as a yellow oil that quickly became a black-green oil upon exposure to the atmosphere with the enol tautomer predominating in NMR: ^1^H NMR (400 MHz, CDCl_3_) δ 6.07 (s, 1H), 3.89 (s, 4H). ^13^C­{^1^H} NMR (101 MHz, CDCl_3_) δ 186.8, 98.4, 30.1; HRMS (ESI) *m*/*z*: [M + H]^+^ calcd for C_5_H_7_Br_2_O_2_ 256.8813; found 256.8806.

#### 1,5-Bis­(phenylthio)­pentane-2,4-dione (**30**)

To a round-bottomed flask containing CHCl_3_ (40 mL) was added thiophenol (3.30 mL, 32.4 mmol). The mixture was cooled in an ice/water bath, and after stirring for 15 min, DBU (5.30 mL, 35.5 mmol) was added dropwise. After stirring for 5 min, **29** (4.18 g, 16.2 mmol) dissolved in CHCl_3_ (10 mL) was added dropwise. The ice bath was removed, and the reaction was allowed to warm to room temperature with stirring overnight. The mixture was diluted with CHCl_3_ (25 mL), washed with water (25 mL), aqueous HCl (1 M, 2 × 25 mL), NaHCO_3_ (25 mL), and brine (25 mL), dried (Na_2_SO_4_), and concentrated. The residue was purified using automated flash chromatography (SiO_2_, 4–6% EtOAc:Hex) to yield **30** (3.92 g, 77%) as a dark-orange oil as a mixture of the dominant symmetric enol and another tautomer. ^1^H NMR (400 MHz, CDCl_3_) δ 7.34–7.19 (m, 13.4H (enol and tautomer)), 5.89 (s, 1H (enol)), 3.93 (s, 0.35H (tautomer)), 3.85 (s, 0.7H (tautomer)), 3.70 (s, 0.7H (tautomer)), 3.58 (s, 4H (enol)); ^13^C­{^1^H} NMR (101 MHz, CDCl_3_) δ 199.2 (tautomer), 189.5 (enol), 134.7 (enol), 130.3 (enol), 129.8 (tautomer), 129.4 (tautomer), 129.2 (enol), 127.4 (tautomer), 127.3 (enol), 98.8 (enol), 51.6 (tautomer), 44.4 (tautomer), 40.4 (enol); HRMS (ESI) *m*/*z*: [M + H]^+^ calcd for C_17_H_17_O_2_S_2_ 317.0664; found 317.0665.

#### Dimethyl 2-hydroxy-4,6-bis­((phenylthio)­methyl)­isophthalate (**32**)

To a flame-dried flask was added Na (0.092 g, 3.8 mmol). After the mixture was purged with nitrogen and cooled in an ice/water bath, CH_3_OH (22 mL) was added slowly with proper ventilation for off-gassing hydrogen. Once the metal had dissolved, **31** (0.510 mL, 3.53 mmol) was added dropwise. The cooling bath was removed, and the reaction mixture was stirred for 5 min, after which diketone **30** (1.06 g, 3.34 mmol) dissolved in CH_3_OH (8 mL) was added dropwise. The mixture was stirred for 3.5 days. The mixture was filtered (filter paper, CH_3_OH), and the filtrate was acidified (pH 1) using aqueous 1 M HCl. The mixture was concentrated, and the resulting oil was dissolved in CH_2_Cl_2_ (20 mL). Water was added (16 mL), and the layers were separated. The aqueous layer was washed with CH_2_Cl_2_ (2 × 20 mL). The organic layers were combined, washed with brine (20 mL), and concentrated. Purification using automated flash chromatography (SiO_2_, 13–26% EtOAc:Hex) gave **32** (1.06 g, 40%, 56% brsm) as a yellow oil: ^1^H NMR (400 MHz, CDCl_3_) δ 11.68 (s, 1H), 7.25–7.18 (m, 10H), 6.55 (s, 1H), 4.12 (s, 4H), 3.90 (s, 6H); ^13^C­{^1^H} NMR (101 MHz, CDCl_3_) δ 169.1, 160.6, 142.5, 135.4, 131.3, 129.1, 127.3, 123.8, 116.6, 52.8, 39.0; HRMS (ESI) *m*/*z*: [M + H]^+^ calcd for C_24_H_23_O_5_S_2_ 455.0987, found 455.0967.

#### Dimethyl 2-methoxy-4,6-bis­((phenylthio)­methyl)­isophthalate (**33**)

A flame-dried flask was charged with K_2_CO_3_ (0.160 g, 1.13 mmol) and purged with nitrogen. Phenol **32** (0.29 g, 0.64 mmol) was dissolved in acetone (24 mL) and added to the flask, followed by a dropwise addition of CH_3_I (0.10 mL, 1.6 mmol). The mixture was refluxed overnight, after which it was cooled to room temperature and concentrated. The residue was suspended in EtOAc (25 mL) with stirring and filtered (filter paper, EtOAc). The filtrate was dried (Na_2_SO_4_) and concentrated to yield **33** (0.296 g, 99%) as a yellow, amorphous solid. ^1^H NMR (400 MHz, CDCl_3_) δ 7.26–7.15 (m, 10H), 6.90 (s, 1H), 4.04 (s, 4H), 3.88 (s, 6H), 3.80 (s, 3H); ^13^C­{^1^H} NMR (101 MHz, CDCl_3_) δ 167.2, 156.0, 139.1, 135.2, 131.1, 129.1, 127.2, 127.1, 127.0, 63.8, 52.7, 37.1; HRMS (ESI) *m*/*z*: [M + H]^+^ calcd for C_25_H_25_O_5_S_2_ 469.1138; found 469.1124.

#### Dimethyl 2-methoxy-4,6-bis­((phenylsulfinyl)­methyl)­isophthalate (**18**)

Compound **33** (1.40 g, 2.99 mmol) was dissolved in a mixture of CH_3_OH (70 mL) and water (10 mL), after which NaIO_4_ (1.59 g, 7.45 mmol) was added, and the mixture was stirred overnight. The mixture was filtered (filter paper, CH_3_OH), and the filtrate was concentrated. The residue was dissolved in EtOAc (60 mL), washed with water (20 mL) and brine (20 mL), dried (Na_2_SO_4_), and concentrated. Purification using automated flash chromatography (SiO_2_, 50–100% EtOAc:Hex) yielded **18** (1.30 g, 87%) as a yellow-white amorphous solid. Because of the two chiral sulfoxide centers, the compound exists as an inseparable 1:1 mixture of the enantiomeric pair and the meso isomer: ^1^H NMR (400 MHz, CDCl_3_) δ 7.44–7.50 (m, 20H), 6.65 (s, 1H), 6.56 (s, 1H), 4.14 (d, *J* = 12.7 Hz, 2H), 4.07 (s, 4H), 3.99 (d, *J* = 12.8 Hz, 2H), 3.90 (s, 6H), 3.89 (s, 6H), 3.79 (s, 3H), 3.77 (s, 3H); ^13^C­{^1^H} NMR (101 MHz, CDCl_3_) δ 166.7, 166.6, 157.0, 156.9, 143.3, 143.1, 132.4, 132.1, 131.60, 131.58, 130.6, 130.3, 129.4, 129.3, 128.7, 128.6, 124.3, 124.2, 63.8, 61.6, 61.3, 52.9 (2C); (ESI) *m*/*z*: [M + H]^+^ calcd for C_25_H_25_O_7_S_2_ 501.1042, found 501.1025.

#### Dimethyl 2-methoxy-4-((phenylsulfinyl)­methyl)-6-((phenylthio)­methyl)­isophthalate (**34**)

Compound **33** (0.126 g, 0.270 mmol) was dissolved in a mixture of CH_3_OH (12 mL) and water (1.5 mL), after which NaIO_4_ (0.056 g, 0.26 mmol) was added, and the mixture was stirred overnight. The mixture was filtered (filter paper, CH_3_OH), and the filtrate was concentrated. Purification using automated flash chromatography (SiO_2_, 33 to 67% EtOAc:Hex) yielded **34** (0.90 g, 70%) as an amorphous off-white solid: ^1^H NMR (400 MHz, CDCl_3_) δ 7.36–7.50 (m, 5H), 7.17–7.29 (m, 5H), 6.74 (s, 1H), 4.04–4.11 (m, 2H), 4.04 (s, 2H), 3.90 (s, 3H), 3.85 (s, 3H), 3.77 (s, 3H); ^13^C­{^1^H} NMR (101 MHz, CDCl_3_) δ 167.0, 166.8, 156.5, 142.9, 139.5, 135.0, 131.6, 131.5, 131.1, 129.4, 129.2, 129.1, 128.7, 128.4, 127.3, 124.3, 63.6, 61.3, 52.7, 52.6, 36.8; HRMS (ESI) *m*/*z*: [M + H]^+^ calcd for C_25_H_25_O_6_S_2_ 485.1093, found 485.1089.

#### Dimethyl 10-hydroxy-9-methoxy-1-oxo-7-((phenylsulfinyl)­methyl)-1*H*-benzo­[*g*]­isochromene-3,8-dicarboxylate (**20**)

A flask charged with THF (0.4 mL) and LiHMDS (1 M, 1.2 mL, 1.2 mmol) was cooled to −78 °C by using a dry ice/acetone bath. After stirring for 10 min, pyrone **7** (0.167 g, 1.08 mmol) dissolved in THF (5 mL) was added dropwise. After the mixture was stirred for 15 min, disulfoxide **18** (0.224 g, 0.447 mmol) dissolved in THF (3 mL) was added dropwise. The mixture was stirred for 45 min at −78 °C and then warmed to room temperature overnight. The mixture was acidified (pH 1) using aqueous HCl (3.24 M, ∼ 6 mL), and the THF was removed under reduced pressure. The aqueous layer was extracted with EtOAc (3 × 10 mL). The organic layers were combined, washed with brine (10 mL), dried (Na_2_SO_4_), and concentrated. Repeated purification of the residue using automated flash chromatography (SiO_2_, 15–25% EtOAc:CHCl_3_) yielded **20** (0.072 g, 32%) as an orange amorphous solid: ^1^H NMR (600 MHz, CDCl_3_) δ 12.96 (s, 1H), 7.53–7.45 (m, 6H), 7.41 (s, 1H), 7.33 (s, 1H), 4.22 (m, 2H), 3.98 (s, 3H), 3.97 (s, 3H), 3.96 (s, 3H); ^13^C­{^1^H} NMR (151 MHz, CDCl_3_) δ 167.4, 166.3, 163.2, 160.5, 157.8, 142.7, 142.2, 140.0, 131.7, 131.3, 130.9, 129.4, 127.7, 126.7, 124.3, 118.4, 117.6, 113.9, 102.2, 64.7, 62.3, 53.3, 52.9; HRMS (ESI) *m*/*z*: [M + H]^+^ calcd for C_25_H_21_O_9_S 497.0888; found 497.0906.

#### Dimethyl 10-hydroxy-9-methoxy-5-(4-methoxybenzyl)-1-oxo-7-((phenylsulfinyl)­methyl)-1*H*-benzo­[*g*]­isochromene-3,8-dicarboxylate (**35**)

A flame-dried flask was charged with anhydrous K_2_CO_3_ (0.200 g, 1.45 mmol), NaI (0.144 g, 0.961 mmol), DMAP (0.004 g, 0.003 mmol), DMF (3 mL), and PMBCl (0.120 mL, 0.663 mmol) followed by **20** (0.330 g, 0.665 mmol) dissolved in DMF (9 mL) in three 3 mL portions. The mixture was heated to 70 °C (oil bath) and stirred for 24 h. Additional PMBCl (0.100 mL, 0.553 mmol) was added, and the stirring mixture was heated at 85 °C (oil bath) and stirred for 12 h. After cooling, the mixture was partitioned between water (30 mL) and EtOAc (75 mL). The organic layer was washed with saturated aqueous NH_4_Cl (25 mL) and brine (25 mL), dried with Na_2_SO_4_, and concentrated. The residue was purified using automated flash chromatography (SiO_2_, 12–50% EtOAc:CH_2_Cl_2_) yielding **35** as an amorphous, light-orange solid: ^1^H NMR (100 MHz, CDCl_3_) δ 13.26 (s, 1H), 7.74 (s, 1H), 7.47 (s, 1H), 7.38–7.55 (m, 5H), 6.69–6.99 (m, 4 H), 4.26–4.37 (m, 2 H), 4.16 (s, 2H), 3.97 (s, 3H), 3.95 (s, 6H), 3.74 (s, 3H). ^13^C­{^1^H} NMR (125 MHz, CDCl_3_) δ 167.3, 166.8, 162.7, 160.6, 158.4, 158.2, 142.9, 142.0, 139.3, 131.6, 131.2, 131.1, 129.5, 129.2, 128.8, 126.9, 124.7, 124.6, 124.4, 118.9, 114.4, 111.1, 102.0, 64.6, 62.4, 55.4, 53.3, 52.9, 32.5; HRMS (ESI) *m*/*z*: [M + H]^+^ calcd for C_33_H_29_O_10_S 617.1481, found 617.1476.

#### Dimethyl 10-((*tert*-butyldimethylsilyl)­oxy)-9-methoxy-1-oxo-7-((phenylsulfinyl)­methyl)-1*H*-benzo­[*g*]­isochromene-3,8-dicarboxylate (**36**)

To a stirring solution of **20** (0.188 g, 0.379 mmol) dissolved in CH_2_Cl_2_ (10 mL) was added DMAP (0.006 g, 0.05 mmol) and *i*-Pr_2_NEt (0.99 mL, 5.7 mmol). The mixture was cooled in an ice bath, and TBSOTf (0.65 mL, 2.8 mmol) was added. The mixture was warmed to rt and stirred for 3 h, after which additional TBSOTf (0.65 mL, 2.8 mmol) was added, and stirring was continued overnight. The mixture was quenched by the addition of saturated aqueous NaHCO_3_ (15 mL) and extracted with CH_2_Cl_2_ (2 × 10 mL). The combined organic layers were dried with Na_2_SO_4_ and concentrated. The residue was purified using automated flash chromatography (SiO_2_, 0–1% EtOAc:CHCl_3_, then SiO_2_, 0–1% EtOAc:CHCl_3_) to give **36** (0.049 g, 21%) as an orange, amorphous solid: ^1^H NMR (4 00 MHz, CDCl_3_) δ 7.43–7.52 (m, 5H), 7.47 (s, 1H), 7.405 (s, 1H), 7.401 (s, 1H) 4.19 (s, 2H), 3.96 (s, 3H), 3.94 (s, 3H), 3.81 (s, 3H), 1.11 (s, 9H), 0.00 (s, 3H), −0.02 (s, 3H); ^13^C­{^1^H} NMR (100 MHz, CDCl_3_) δ 167.6, 161.1, 158.8, 157.8, 157.5, 143.2, 142.9, 139.1, 133.1, 131.6, 130.1, 129.3, 127.1, 126.2, 124.2, 122.4, 120.3, 112.3, 111.2, 63.9, 62.4, 53.0, 52.8, 26.3, 18.7, −4.22, −4.24; HRMS (ESI) *m*/*z*: [M + Na]^+^ calcd for C_31_H_34_O_9_SSiNa 633.1590, found 633.1581.

## Supplementary Material



## Data Availability

The data underlying this study are available in the published article and its Supporting Information.

## Notes

Experimental protocols for the preparation of known compounds **6**–**8** and **13**; and ^1^H and ^13^C­{^1^H} NMR spectra (PDF)
