# Supplementary material for: Annulation Methods toward the Total Synthesis of Thermorubin: Construction of the AB and BCD Ring Systems
Source: J Org Chem. 2025 Jul 29;90(31):11207–15. doi: 10.1021/acs.joc.5c01129 (PMC12340950; doi:10.1021/acs.joc.5c01129)
Supplement: Supplementary file 1 [file jo5c01129_si_001.pdf]

Supporting Information

Annulation methods toward the total synthesis of thermorubin: Construction of the AB and BCD ring systems

Authors: Zachary A. Kohanov[a,b] and Andrew N. Lowell[a,b,c,*]

[a]Department of Chemistry, Virginia Polytechnic Institute and State University (Virginia Tech), Blacksburg, VA 24061, USA

[b]Center for Emerging, Zoonotic, and Arthropod-borne Pathogens, Virginia Polytechnic Institute and State University (Virginia Tech), Blacksburg, VA 24061, United States

[c]Faculty of Health Sciences, Virginia Polytechnic Institute and State University (Virginia Tech), Blacksburg, VA 24061, United States

alowell@vt.edu

Table of Contents

Title Page S1

Table of Contents S2

Preparation of **6** and **8** S3–S4

Preparation of **13** S4–S5

Preparation of **7** S5–S6

^{1}H and ^{13}C NMR Spectra S7–S24

References S25

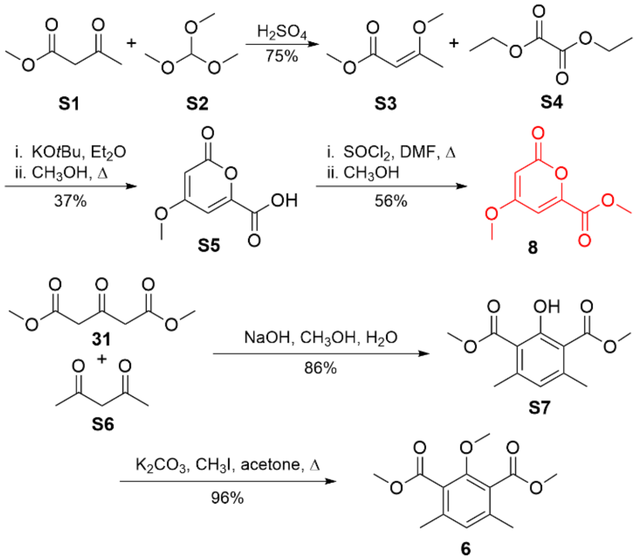

Scheme S1. Synthesis of known precursors **8** and **6**.

Methyl 3-methoxybut-2-enoate (S3). The following reaction was carried out in an analogous manner to the published procedure.[1] To a round-bottomed flask charged with methyl acetoacetate (**S1**, 30.21 g, 260.2 mmol) and trimethyl orthoformate (**S2**, 28.6 mL, 261 mmol) was added concentrated H_2SO_4 (0.45 mL, 8.1mmol). After stirring overnight, the reaction was quenched by the addition of isoquinoline (1.0 mL, 8.4 mmol). The resulting mixture was distilled under reduced pressure and further purified by elution through a pad of silica gel (10% EtOAc/hexanes) to yield **S3** (25.48 g, 75%) as a clear and colorless oil. Spectral data were in accord with those previously reported.[2]

4-Methoxy-2-oxo-2H-pyran-6-carboxylic acid (S5). The following reaction was carried out in an analogous manner to the published procedure.[3] To a flask charged with *t*-BuOK (8.04 g, 71.7 mmol) and diethyl ether (120 mL) under an atmosphere of dry nitrogen was added diethyl oxalate (**S4**, 5.24 g, 35.9 mmol). After the bright-yellow solution had stirred for 15 minutes, methyl (*Z*)-3-methoxybut-2-enoate (**S3**, 4.60 g, 35.3 mmol) was added dropwise to the reaction, and it was left to stir overnight. The precipitate was collected (filter paper) and mixed with methanol (125 mL). After refluxing overnight, the mixture was concentrated (N_2 stream) and the resulting material dissolved in a minimal amount of water. The solution was acidified by the dropwise addition of concentrated H_2SO_4 until a solid formed. The solid was collected by filtration and dried under reduced pressure to yield **S5** as a light brown amorphous solid (2.20 g, 37%): ^1H NMR (400 MHz, CD_3OD) δ 6.75 (d, J = 2.3 Hz, 1H), 5.71 (d, J = 2.3 Hz, 1H), 3.88 (s, 3H); $^{13}C\{^1H\}$ NMR (101 MHz, CD_3OD) δ 173.3, 166.7, 165.3, 157.6, 105.1, 91.7, 57.1; HRMS (ESI) m/z: $[M + H]^+$ Calcd for $C_7H_7O_5$ 171.0293; found 171.0289.

Methyl 4-methoxy-2-oxo-2H-pyran-6-carboxylate (8). To a flask containing acid **S5** (0.278 g, 1.63 mmol) was slowly added $SOCl_2$ (3.0 mL, 41 mmol). Once the reaction vapor evolution had ceased, DMF (0.010 mL, 0.14 mmol) was added, and the reaction was heated to reflux (oil bath) for 2 h. After residual solvent was removed via distillation, anhydrous methanol (5 mL) was added and the reaction was stirred for 24 h. The mixture was filtered (filter paper) the solid was and dried under reduced pressure. The solid material was purified using automated flash chromatography (SiO_2, 0% to 1% EtOAc:$CHCl_3$) to yield **8** as a white-brown amorphous solid (0.169 g, 56%): ^1H NMR (400 MHz, $CDCl_3$) δ 6.87 (d, J = 2.3 Hz, 1H), 5.69 (d, J = 2.3 Hz, 1H),

3.93 (s, 3H), 3.86 (s, 3H); $^{13}C\{^1H\}$ NMR (101 MHz, $CDCl_3$) δ 169.5, 162.2, 159.9, 149.1, 107.8, 93.6, 56.6, 53.4; HRMS (ESI) m/z: $[M + H]^+$ Calcd for $C_8H_9O_5$ 185.0450; found 185.0444.

Dimethyl 2-hydroxy-4,6-dimethylisophthalate (S7). To a flask containing acetylacetone (**S6**, 10.0 g, 0.100 mmol) was added a solution of NaOH (100 mL, 0.1 M) and methanol (50 mL). Dimethyl-1,3-acetonedicarboxylate (**31**, 17.4 g, 0.100 mmol) dissolved in methanol (50 mL) was added. The reaction was stirred for 24 h at rt, at which point a white solid had formed. The reaction was diluted with water (50 mL), filtered (filter paper), and the precipitate washed with water (50 mL). The solid was dried under vacuum to yield **S8** as a white amorphous solid (20.5 g, 86%). Spectra data were in accordance with those previously published.[4]

Dimethyl 2-methoxy-4,6-dimethylisophthalate (6). To a round-bottomed flask charged with anhydrous K_2CO_3 (14.1 g, 102 mmol) and dry acetone (230 mL) under an atmosphere of dry nitrogen was added **S7** (20.2 g, 84.9 mmol). To the stirring mixture was added CH_3I (10.0 mL, 160 mmol), and the mixture was heated to reflux (oil bath) overnight. After cooling, the mixture was filtered (filter paper, acetone) and the filtrate was diluted with Et_2O (250 mL). The filtrate was washed with a saturated aqueous Na_2CO_3 solution (2 x 80 mL) and brine (80 mL), dried (Na_2SO_4), and concentrated to yield **6** (20.5 g, 96%) as a white amorphous solid. Spectra data were in accordance with that previously published.[5]

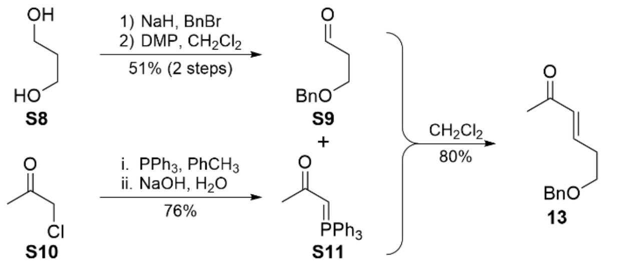

Scheme S2. Synthesis of unsaturated ketone **13**.

3-(Benzyloxy)propanal (S9). The following reaction was carried out in an analogous manner to the published procedure.[6] To a round-bottomed flask containing 1,3-propanediol (**S8**, 6.00 mL, 83.6 mmol) cooled in an ice/water bath was added NaH (0.483 g, 12.1 mmol) in portions. After stirring cold for 15 minutes, BnBr (3.02 g, 17.7 mmol) was added dropwise. The mixture was slowly warmed to rt and then heated at reflux (oil bath) for 16 h. After cooling, water (10 mL) was added and the mixture was stirred for 15 min. The mixture was extracted with CH_2Cl_2 (3 x 25 mL), and the combined organic layers were washed with water (3 x 25 mL), dried (Na_2SO_4), and concentrated, with the alcohol residue being carried onto the next step without further purification.

The following reaction was carried out in an analogous manner to the published procedure.[7] Impure 3-(benzyloxy)propan-1-ol was dissolved in CH_2Cl_2 (36 mL). Dess-Martin periodinane (5.85 g, 20.4 mmol) was added, and the mixture was stirred for 2 h. The mixture was filtered (filter paper, CH_2Cl_2) and the filtrate was washed with water (12 mL), dried (Na_2SO_4), and concentrated. The residue was purified using automated flash chromatography (SiO_2, EtOAc:Hex 25 % to 60 %) to yield **S9** (1.47 g, 51%) as a slightly yellow, clear oil. Spectral data were in accord with those previously reported.[8]

1-(Triphenylphosphoranylidene)-2-propanone (S11). The following reaction was carried out in an analogous manner to the published procedure.[9,10] Triphenyl phosphine (8.30 g, 31.5 mmol) was dissolved in benzene (10 mL). The reaction vessel was purged with nitrogen, and

chloroacetone (**S10**, 2.50 mL, 31.0 mmol) was added. The mixture was stirred overnight, at which point the solid material was removed by filtration (filter paper) and rinsed with benzene (10 mL). The solid material was dissolved in water (50 mL) and basified (pH 10) by the dropwise addition of NaOH (5 M). The resulting precipitate was collected by filtration, dissolved in CH_2Cl_2, dried (Na_2SO_4), and concentrated to yield **S11** (7.6 g, 76%) as a white amorphous solid. Spectral data was in accord with those previously reported.[11]

6-(Benzyloxy)hex-3-en-2-one (13). To a stirring solution of **S11** (0.504 g, 1.58 mmol) dissolved in CH_2Cl_2 (12 mL) was added **S9** (0.204 g, 1.24 mmol) dissolved in CH_2Cl_2 (12 mL). After stirring overnight, the mixture was concentrated and the residue dissolved in Et_2O (30 mL). The mixture was filtered (filter paper, Et_2O) to remove the triphenylphosphine oxide and the filtrate was concentrated. Purification of the residue using automated flash chromatography (SiO_2, 15% to 25% EtOAc:Hex) resulted in **13** (0.203 g, 80%) as a clear and colorless oil. Spectral data were in accord with those previously reported.[12]

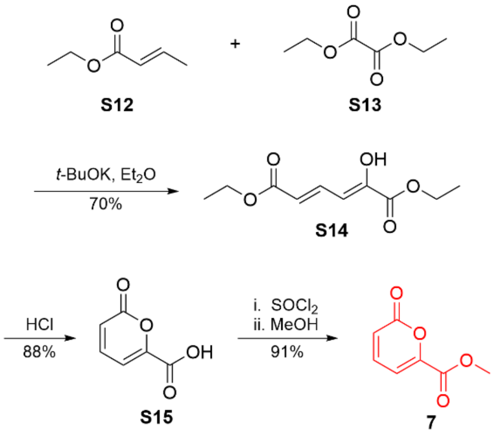

Scheme S3. Formation of pyrone **7**.[13]

6-Ethyl 1-methyl (2*Z*,4*E*)-2-hydroxyhexa-2,4-dienedioate (S14). The following reaction was carried out in an analogous manner to the published procedure.[13] To a flame-dried flask was added *t*-BuOK (24.7 g, 0.220 mol) and Et_2O (86 mL). The flask was purged with nitrogen, and after the mixture had cooled to 0 °C (cryocool), diethyl oxalate (**S13**, 27.1 mL, 0.200 mol) dissolved in Et_2O (16 mL) was added dropwise over 15 min, followed by ethyl crotonate (**S12**, 24.9 mL, 0.200 mol) in a dropwise fashion. The mixture was stirred at 4 °C (cryocool) overnight, after which the reaction was filtered, and the precipitate washed with Et_2O (100 mL). The yellow-orange precipitate was dissolved in cold water (750 mL) and 50% aqueous acetic acid (35 mL) was added. Filtration (water) and drying of the precipitate resulted in **S14** (30 g, 70%) as an amorphous yellow solid. Spectra data were in accord with those previously reported.[14]

2-oxo-2*H*-pyran-6-carboxylic acid (S15). The following reaction was carried out in an analogous manner to the published procedure.[13] Dienedioate (**S14**, 11.65 g, 54.39 mmol) was dissolved in concentrated HCl (325 mL), heated to reflux, and stirred for 8 h. The mixture was cooled to rt and then cooled at -20 °C for 4 h. The precipitate was collected by filtration and the filtrate stored at -20 °C for 12 h, after which additional precipitate was collected. Drying of the combined precipitates yielded **S15** (6.71 g, 88%) as an amorphous gold-yellow solid. Spectra data were in accord with those previously reported.[14]

Methyl 2-oxo-2*H*-pyran-6-carboxylate (7). The following reaction was carried out in an analogous manner to the published procedure.[13] Pyrone carboxylic acid **S15** (0.515 g, 3.68 mmol) was dissolved in $SOCl_2$ (5.0 mL, 69 mmol) and a catalytic amount of DMF (0.015 mL, 0.19 mmol) was added. The reaction was heated to reflux and stirred for 15 h, after which the excess solvent was removed via distillation. The resulting acid chloride was dissolved in methanol (5 mL) and the mixture was stirred at rt for 1 h. The resulting solid material was filtered to give **7** (0.516 g, 91%) as an amorphous beige solid with spectral data in accord with those previously reported.[13] ^1H NMR (400 MHz, $CDCl_3$) δ 7.41 (dd, J = 9.4, 6.6 Hz, 1H), 7.10 (dd, J = 6.5, 1.0 Hz, 1H), 6.55 (dd, J = 9.4, 1.0 Hz, 1H), 3.94 (s, 3H); $^{13}C\{^1H\}$ NMR (101 MHz, $CDCl_3$) δ 159.8, 159.6, 149.4, 141.7, 121.1, 109.9, 53.1. HRMS (ESI) calcd for $C_7H_7O_4$ $[M+H]^+$ 155.0344, found 155.0343.

Figure S1: ^{1}H NMR of **S5** (400 MHz, CD_3OD) plus trace acetic acid.

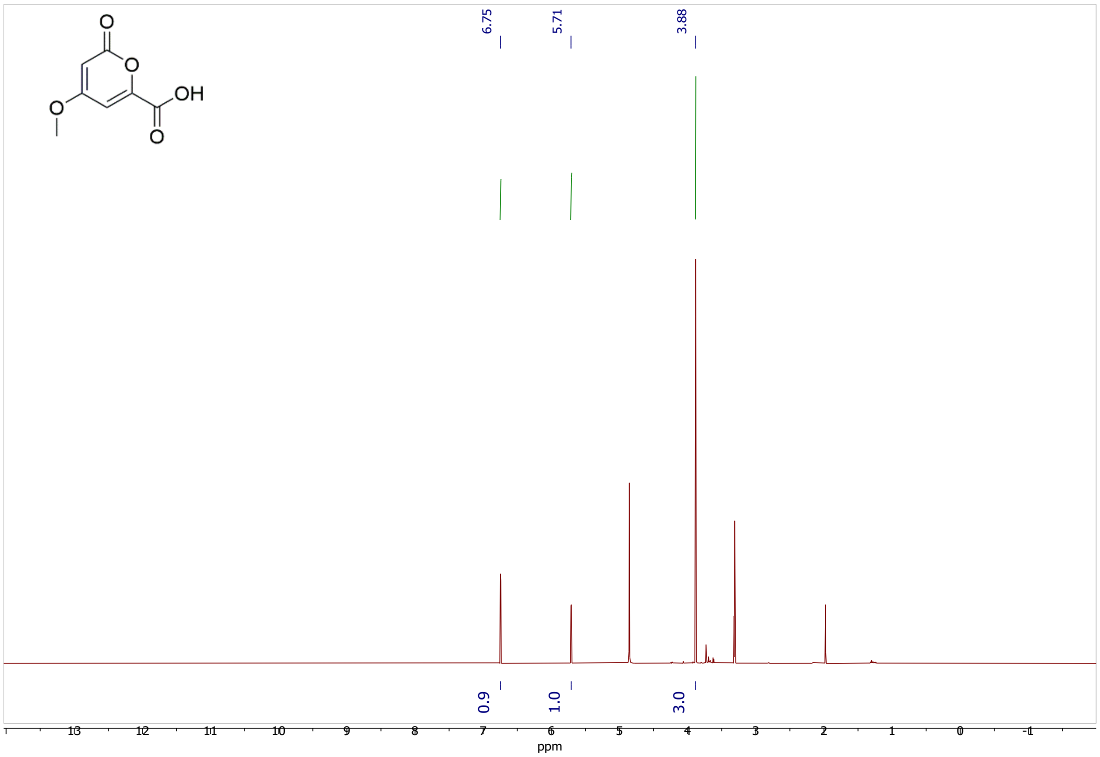

Figure S2: $^{13}C\{^{1}H\}$ NMR of **S5** (101 MHz, CD_3OD).

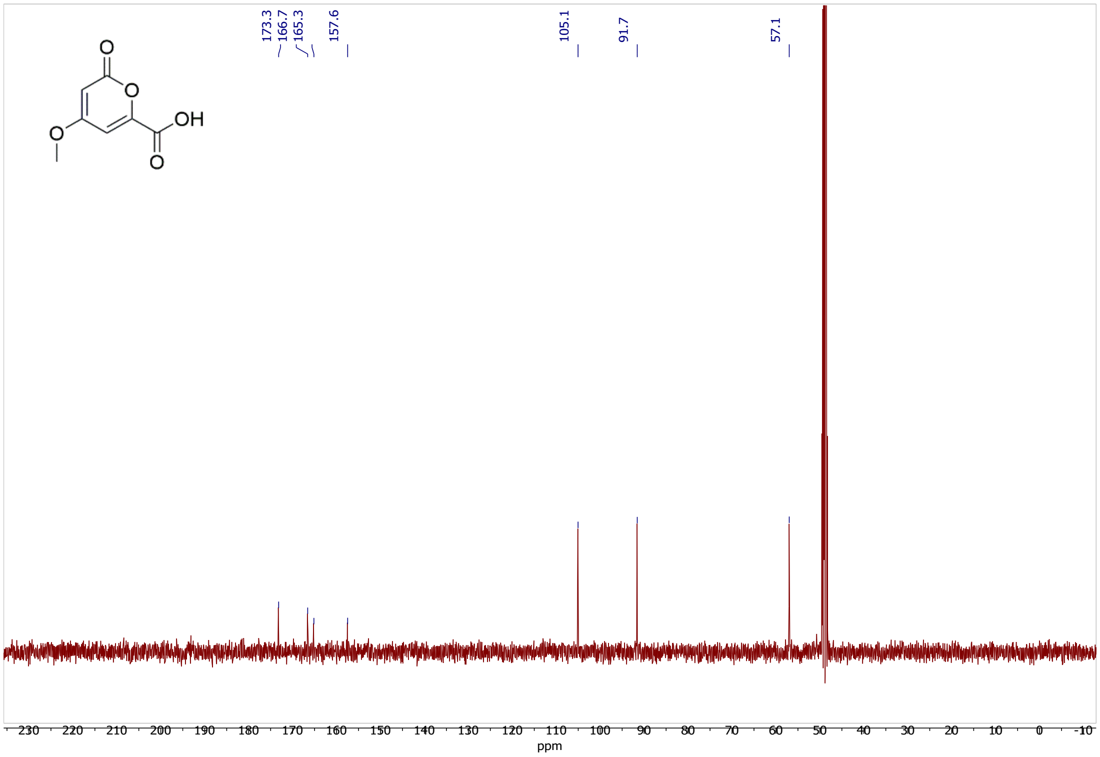

Figure S3: ^{1}H NMR of **8** (400 MHz, $CDCl_3$).

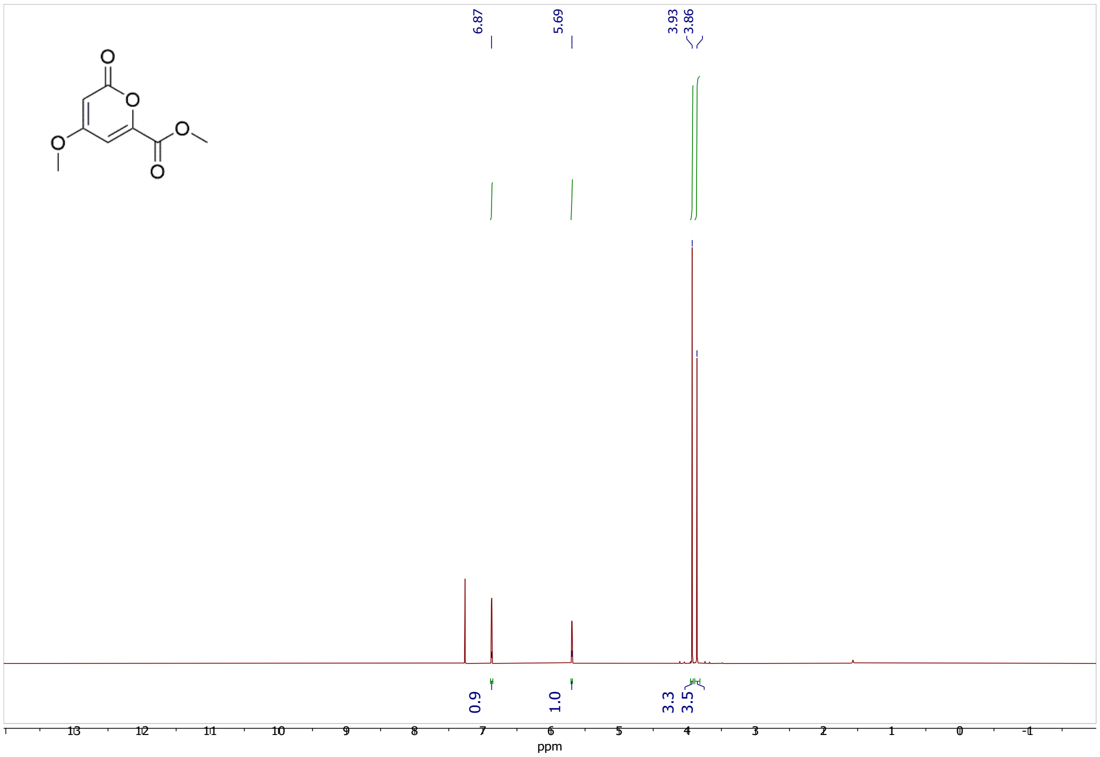

Figure S4: $^{13}C\{^{1}H\}$ NMR of **8** (101 MHz, $CDCl_3$).

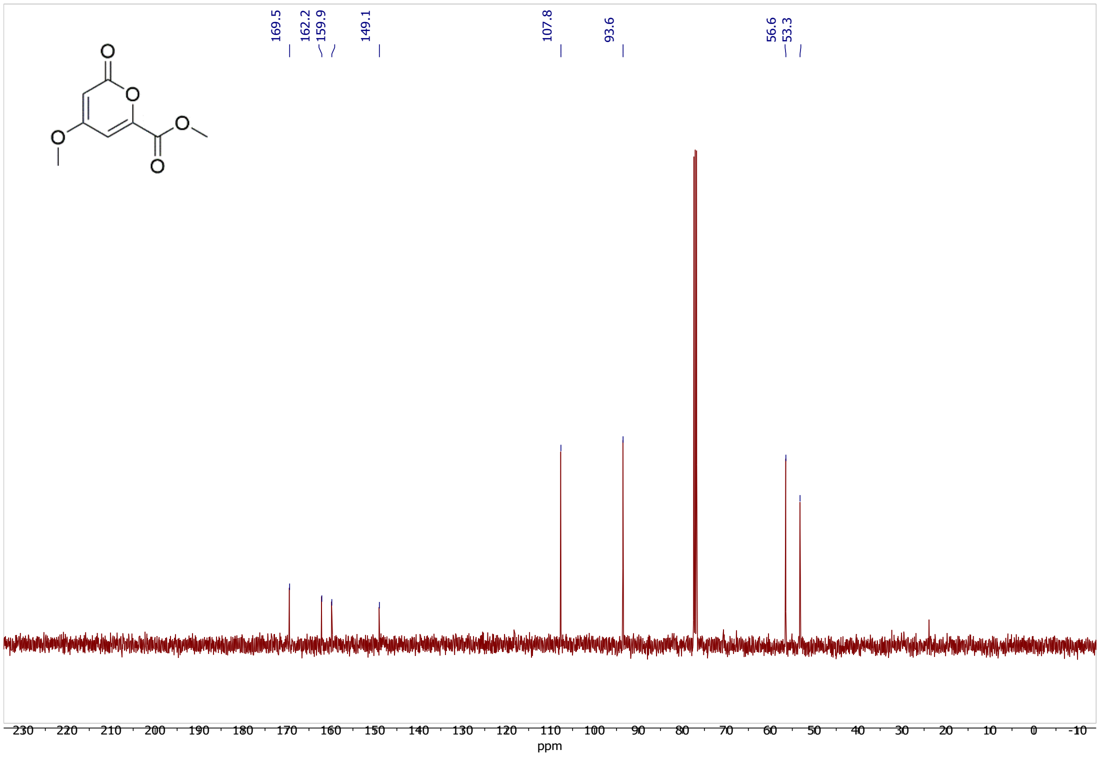

Figure S5: ^{1}H NMR of **12** (400 MHz, $CDCl_3$).

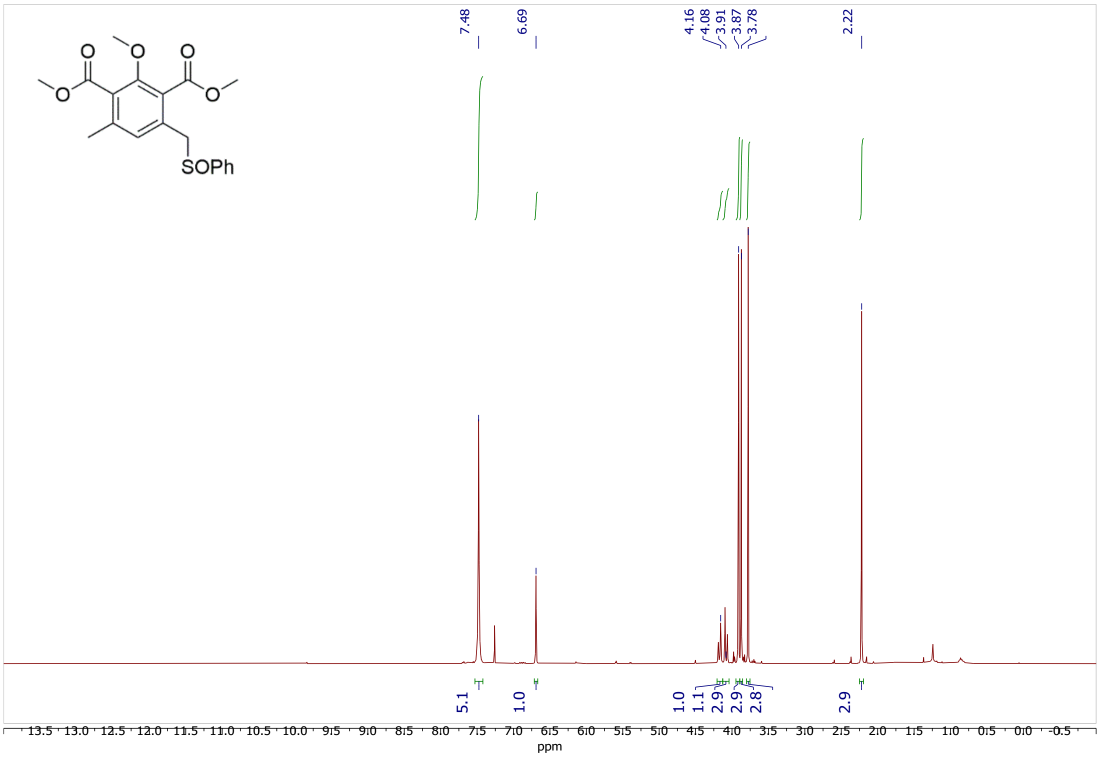

Figure S6: $^{13}C\{^{1}H\}$ NMR of **12** (101 MHz, $CDCl_3$).

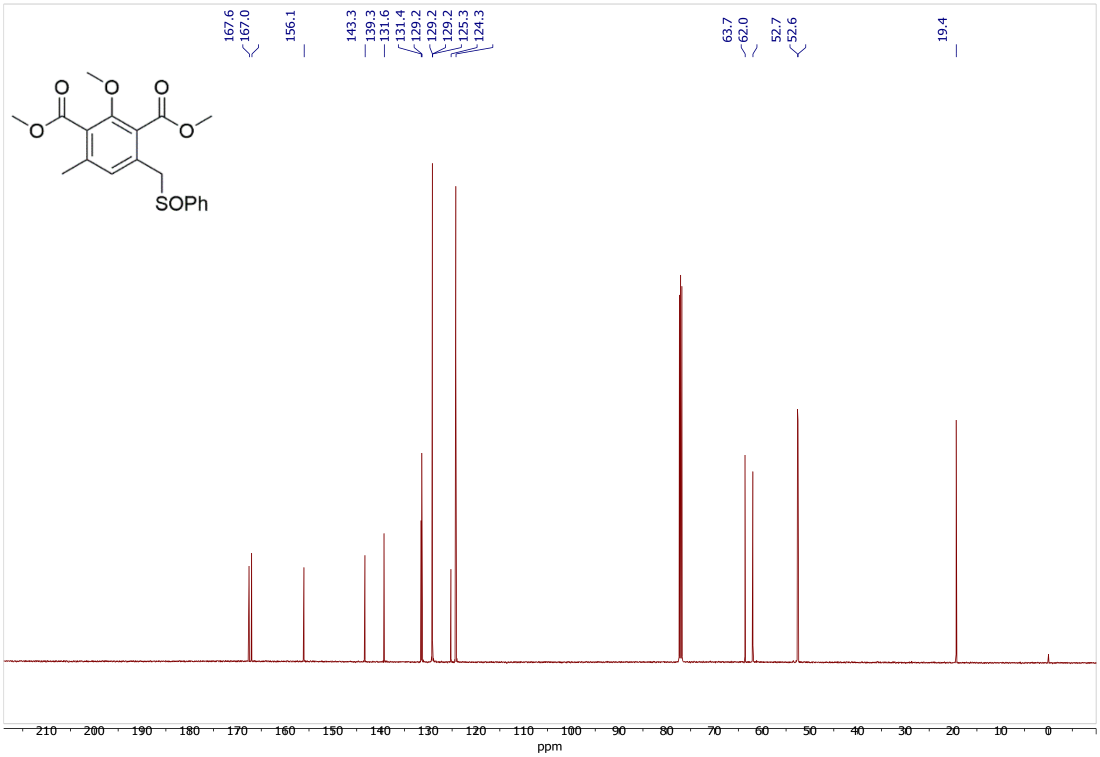

Figure S7: ^{1}H NMR of **14** (400 MHz, $CDCl_3$) plus trace acetic acid.

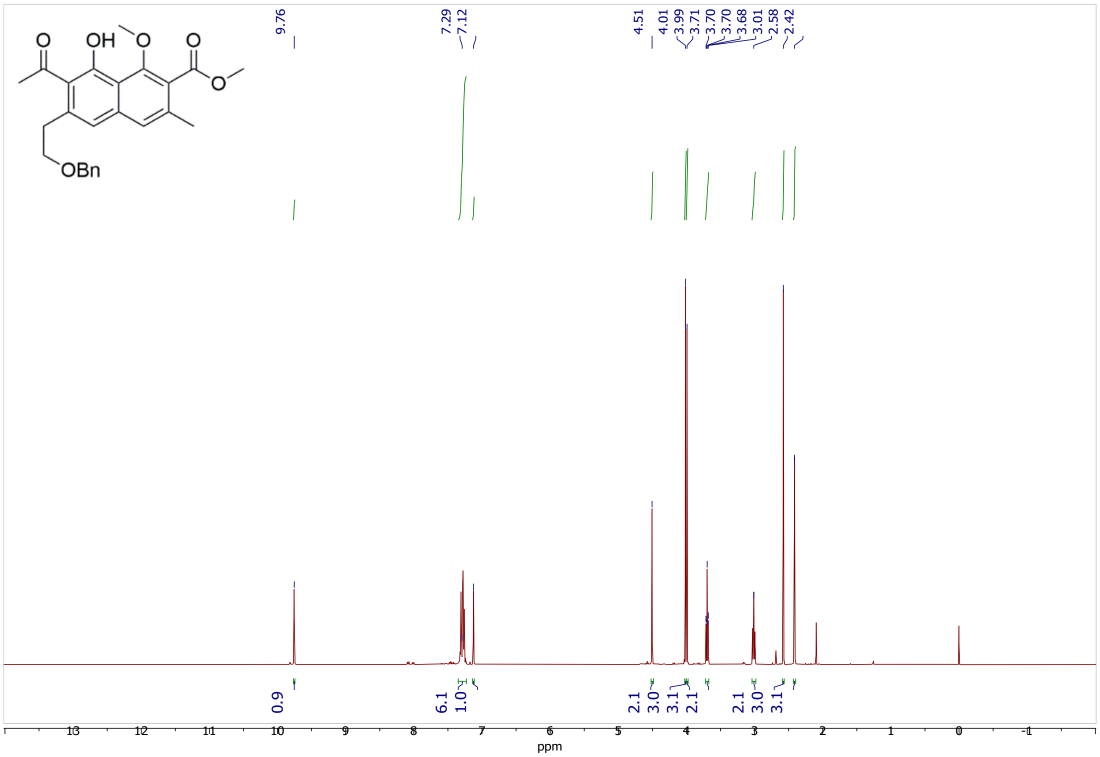

Figure S8: $^{13}C\{^{1}H\}$ NMR of **14** (101 MHz, $CDCl_3$).

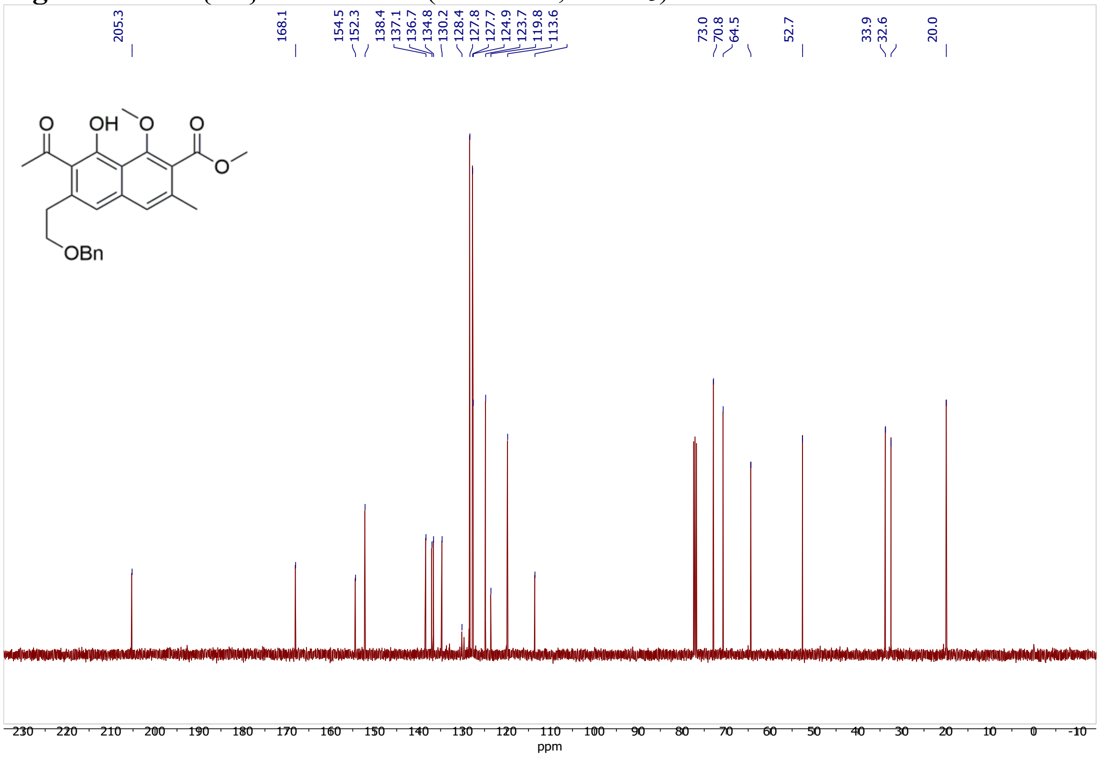

Figure S9: ^{1}H NMR of **15** (400 MHz, $CDCl_3$) plus trace acetone, acetic acid, grease.

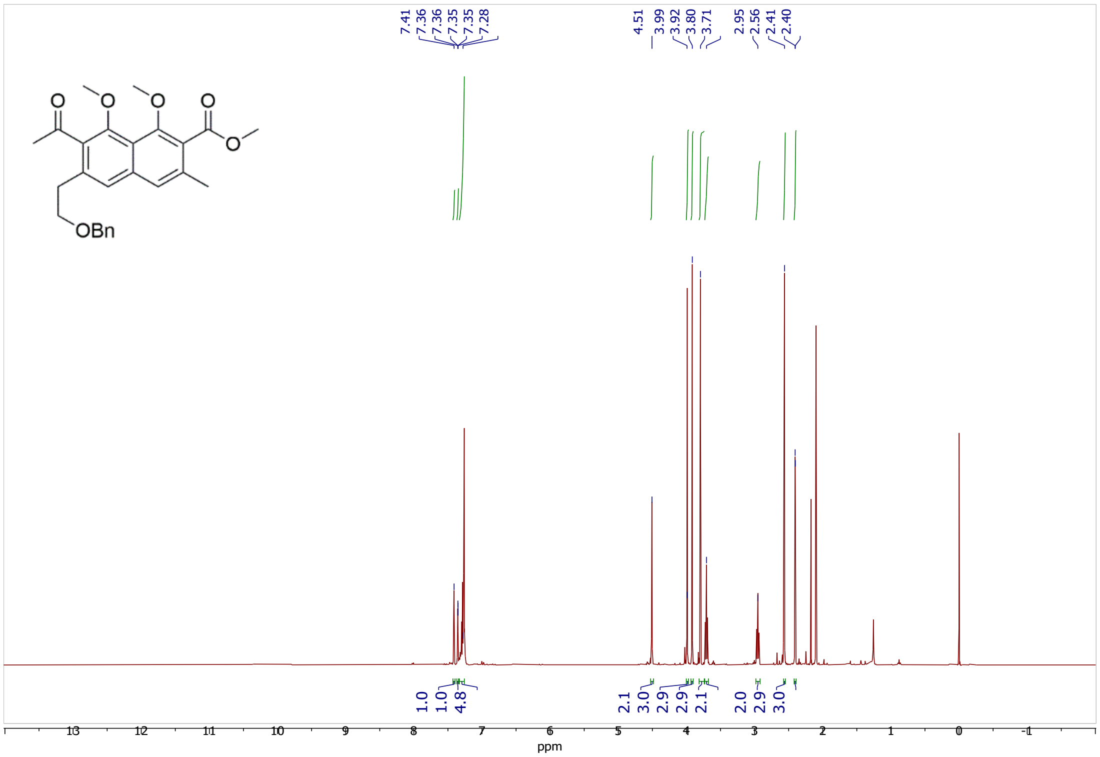

Figure S10: $^{13}C\{^{1}H\}$ NMR of **15** (101 MHz, $CDCl_3$) plus trace acetone, acetic acid.

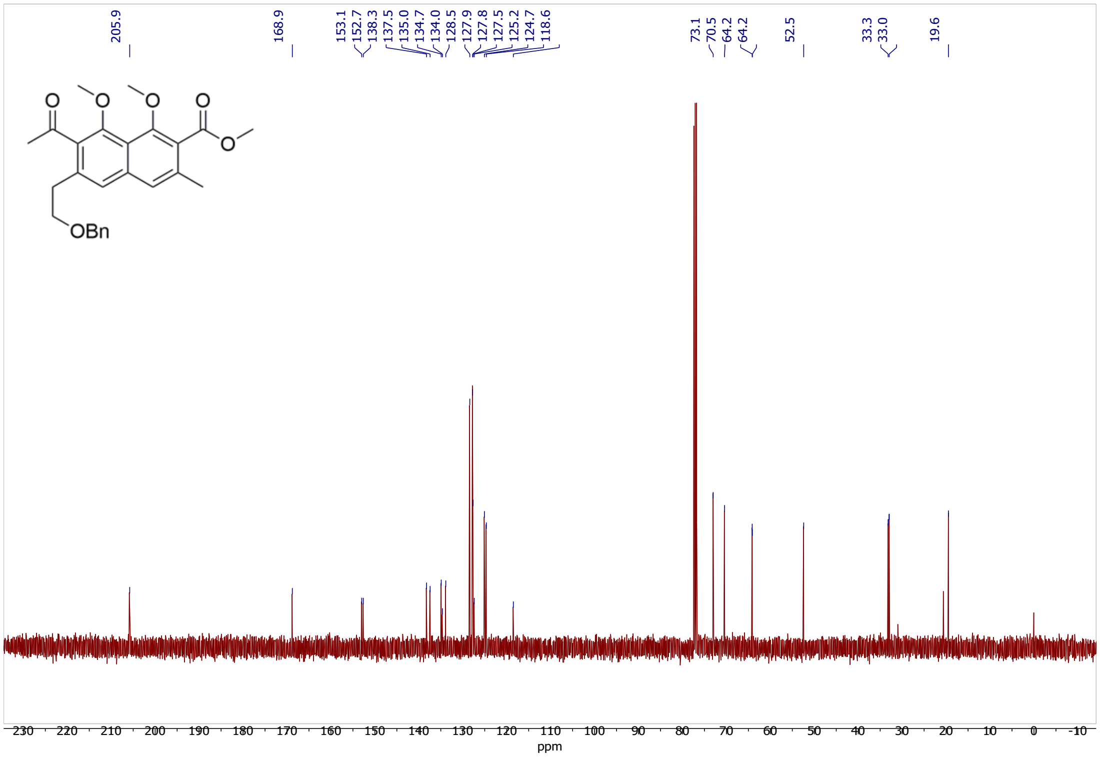

Figure S11: ^{1}H NMR of **7** (400 MHz, $CDCl_3$).

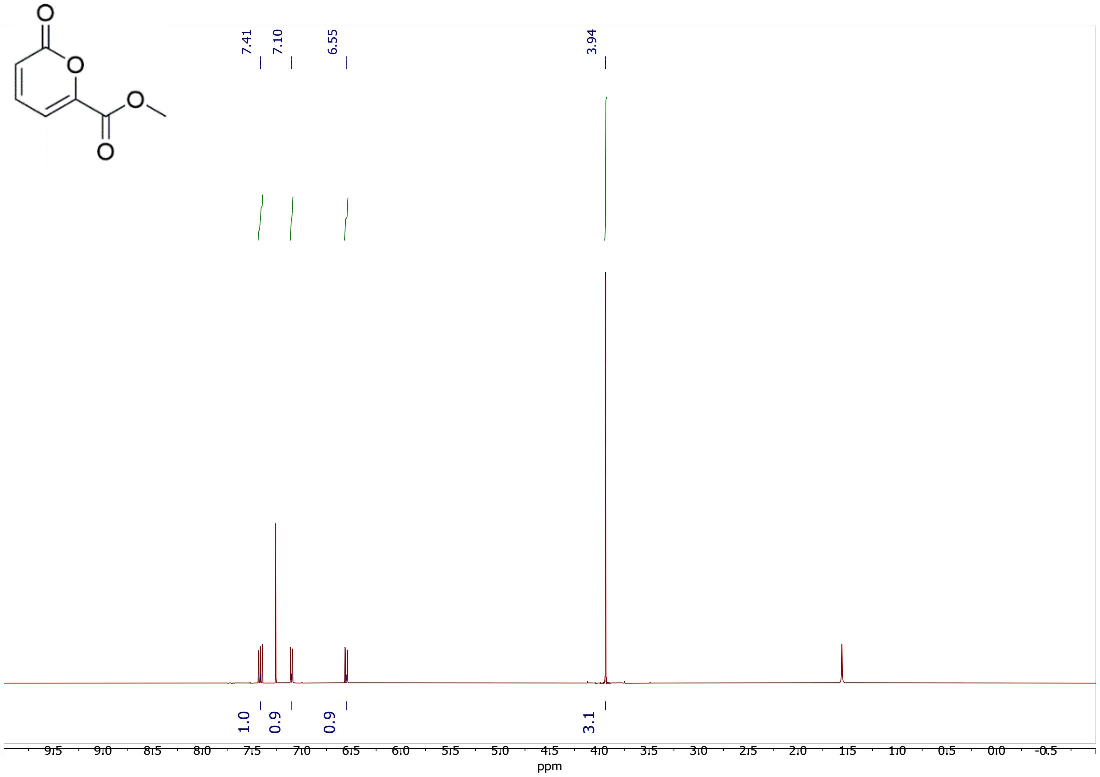

Figure S12: $^{13}C\{^{1}H\}$ NMR of **7** (101 MHz, $CDCl_3$).

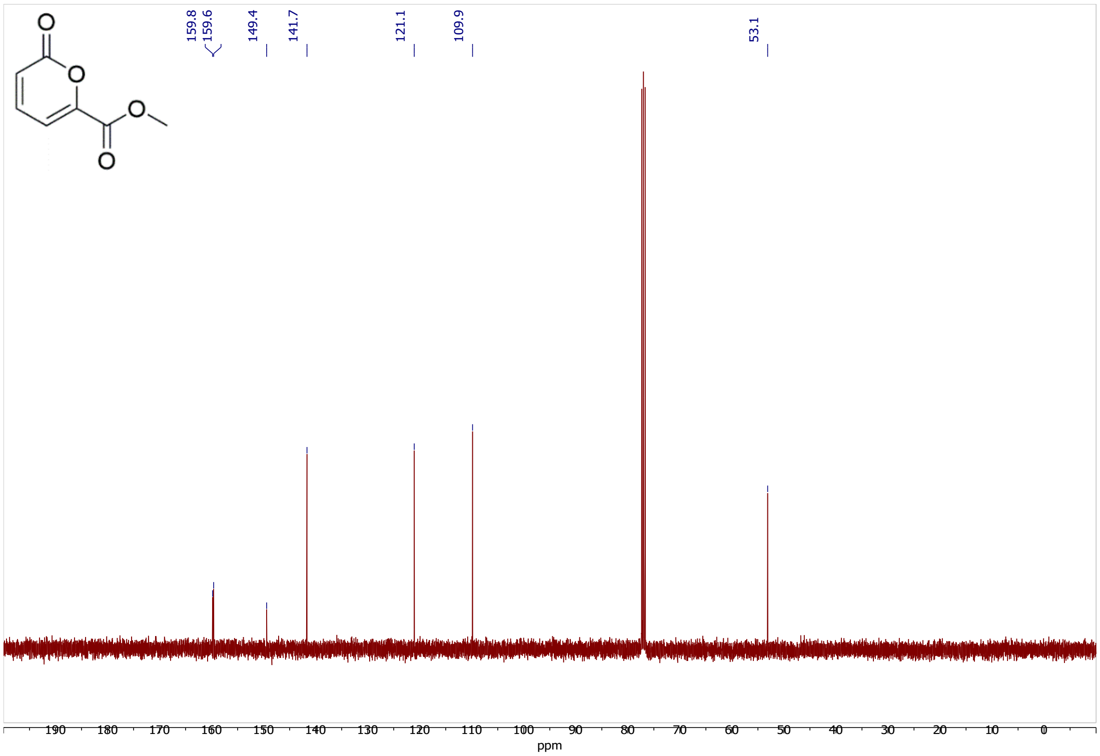

Figure S13: ^{1}H NMR of **19** (400 MHz, $CDCl_3$).

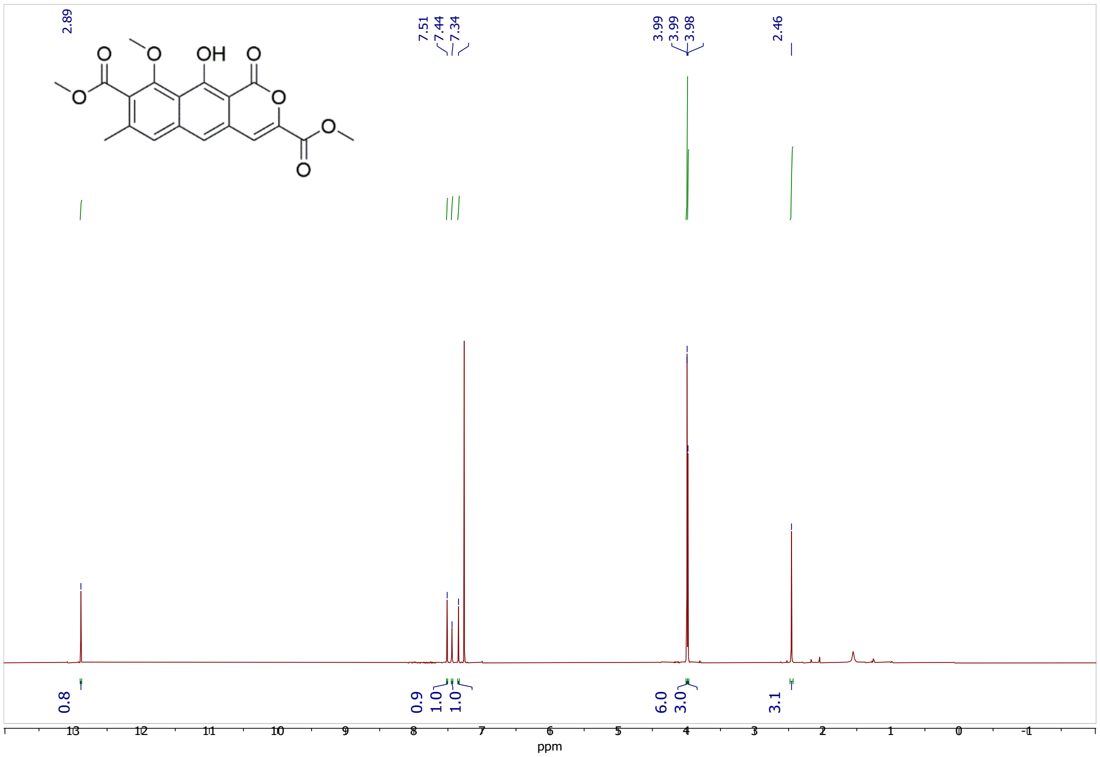

Figure S14: $^{13}C\{^{1}H\}$ NMR of **19** (101 MHz, $CDCl_3$).

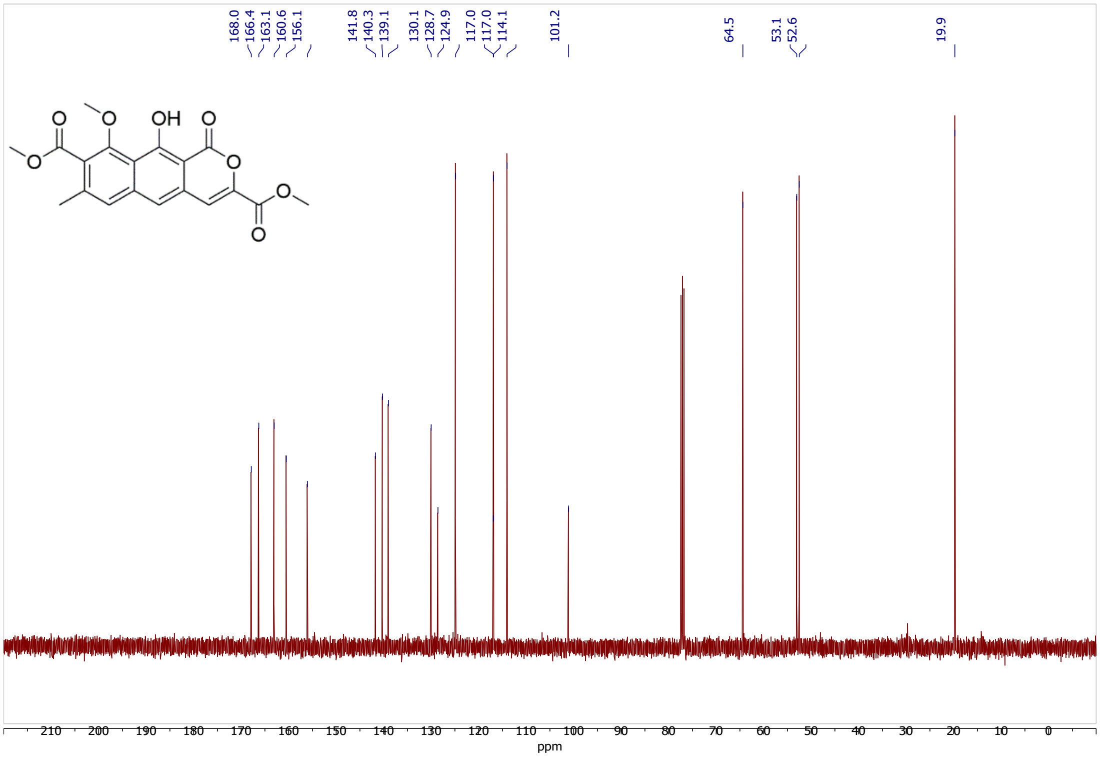

Figure S15: ^{1}H NMR of **21** (400 MHz, $CDCl_3$).

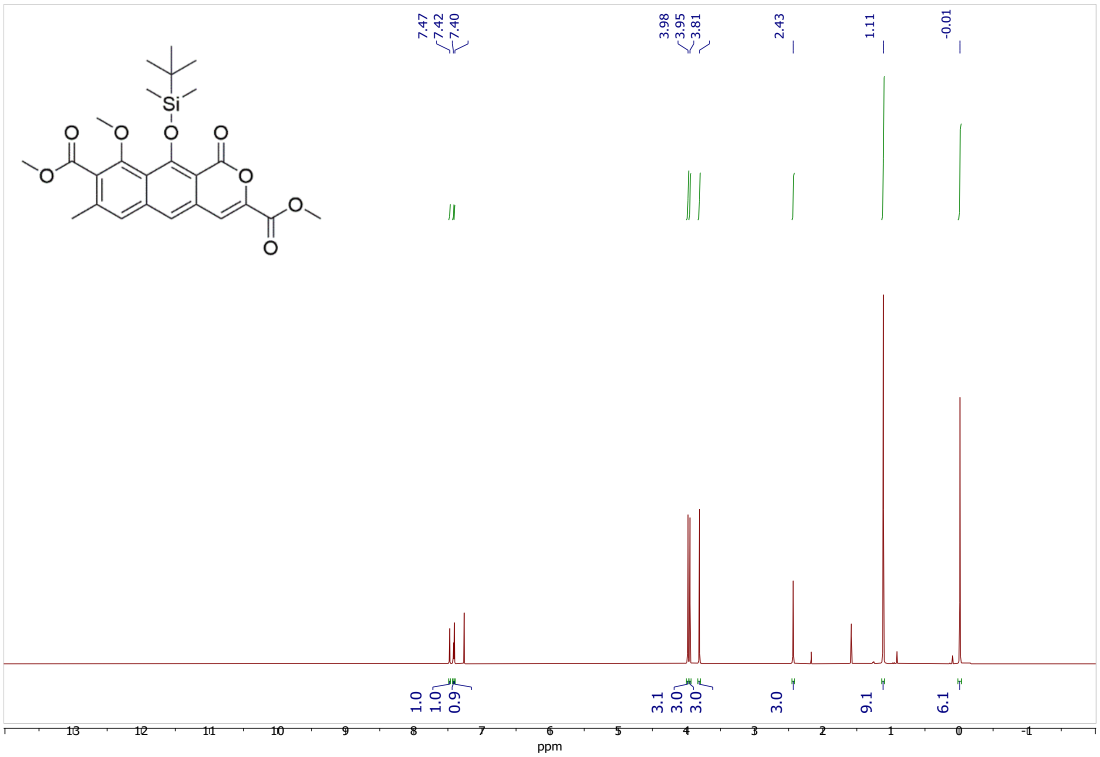

Figure S16: $^{13}C\{^{1}H\}$ NMR of **21** (101 MHz, $CDCl_3$).

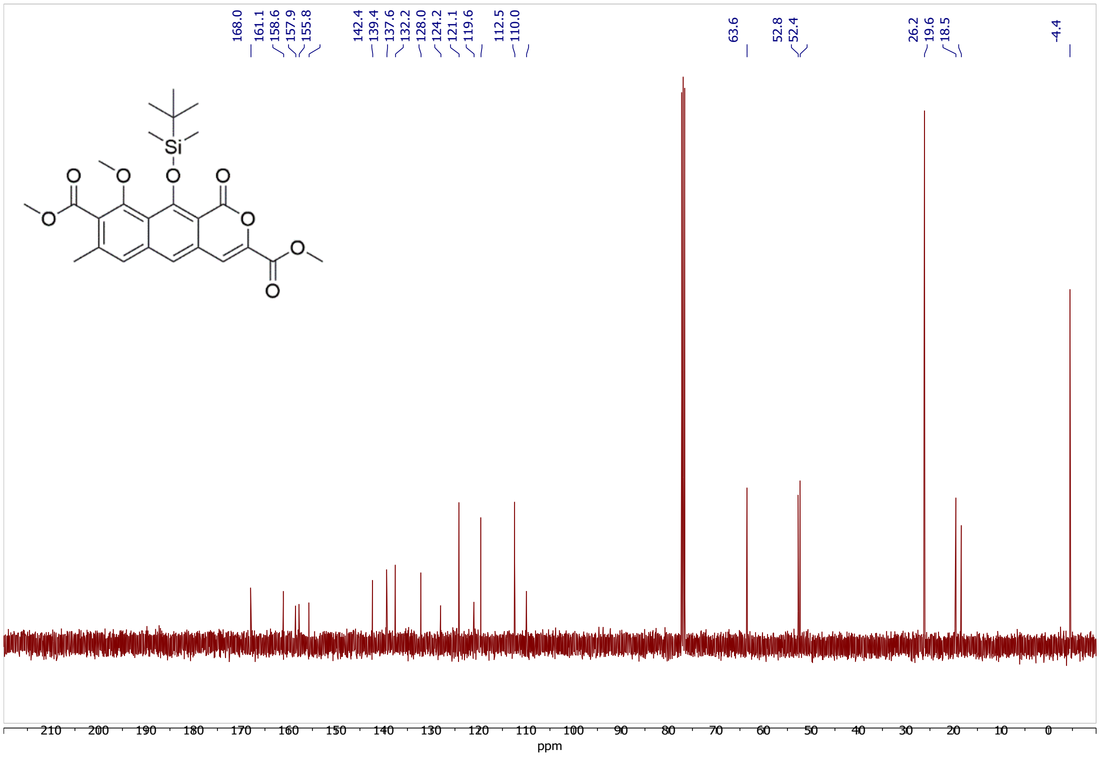

Figure S17: ^{1}H NMR of **28** (400 MHz, $CDCl_3$) plus trace Et_2O.

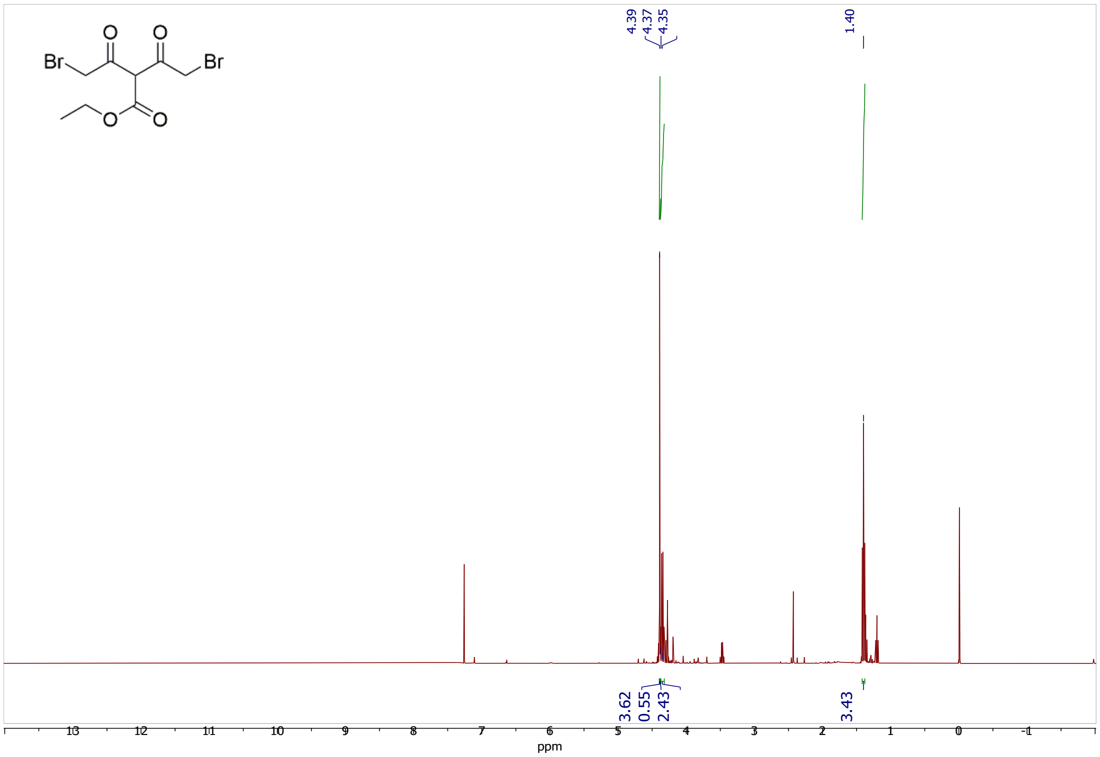

Figure S18: $^{13}C\{^{1}H\}$ NMR of **28** (101 MHz, $CDCl_3$).

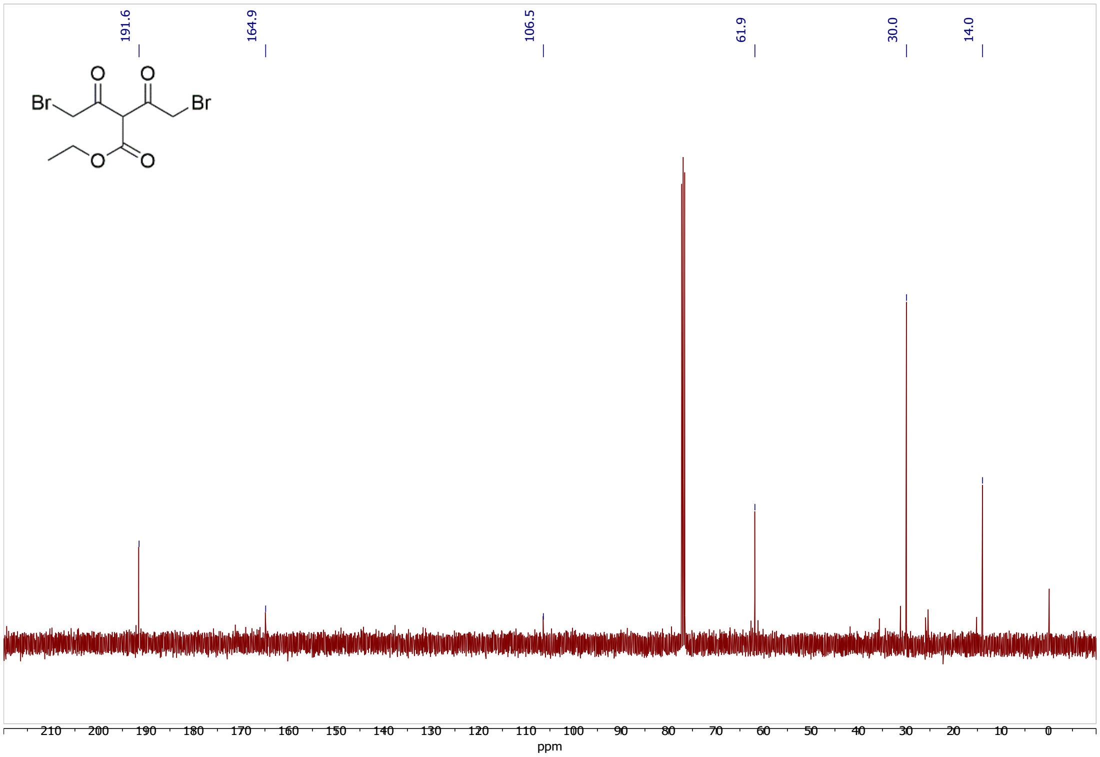

Figure S19: ^{1}H NMR of **29** (400 MHz, $CDCl_3$).

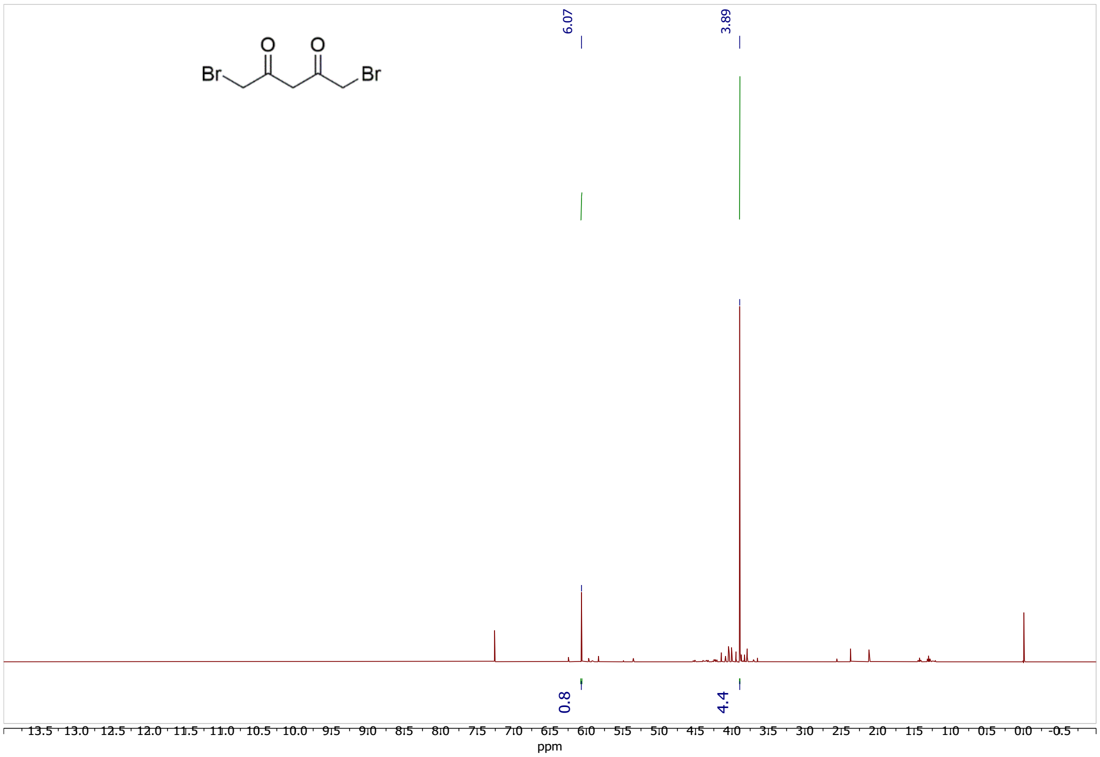

Figure S20: $^{13}C\{^1H\}$ NMR of **29** (101 MHz, $CDCl_3$).

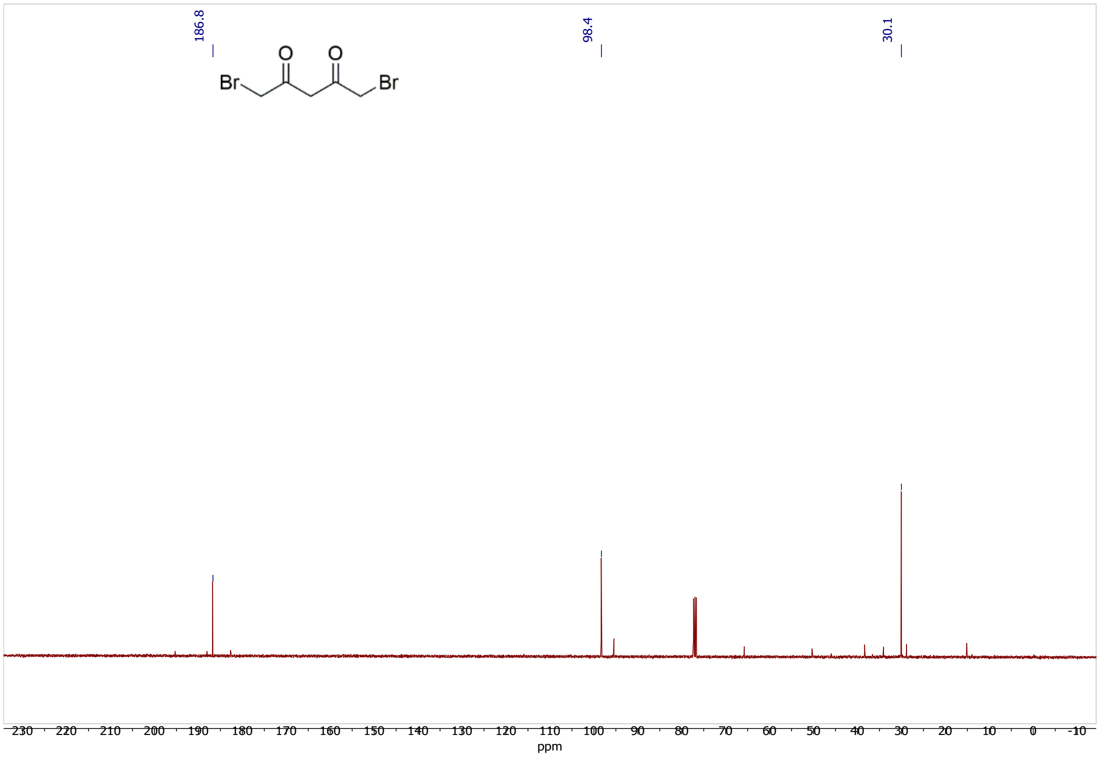

Figure S21: ^{1}H NMR of **30** (400 MHz, $CDCl_3$)

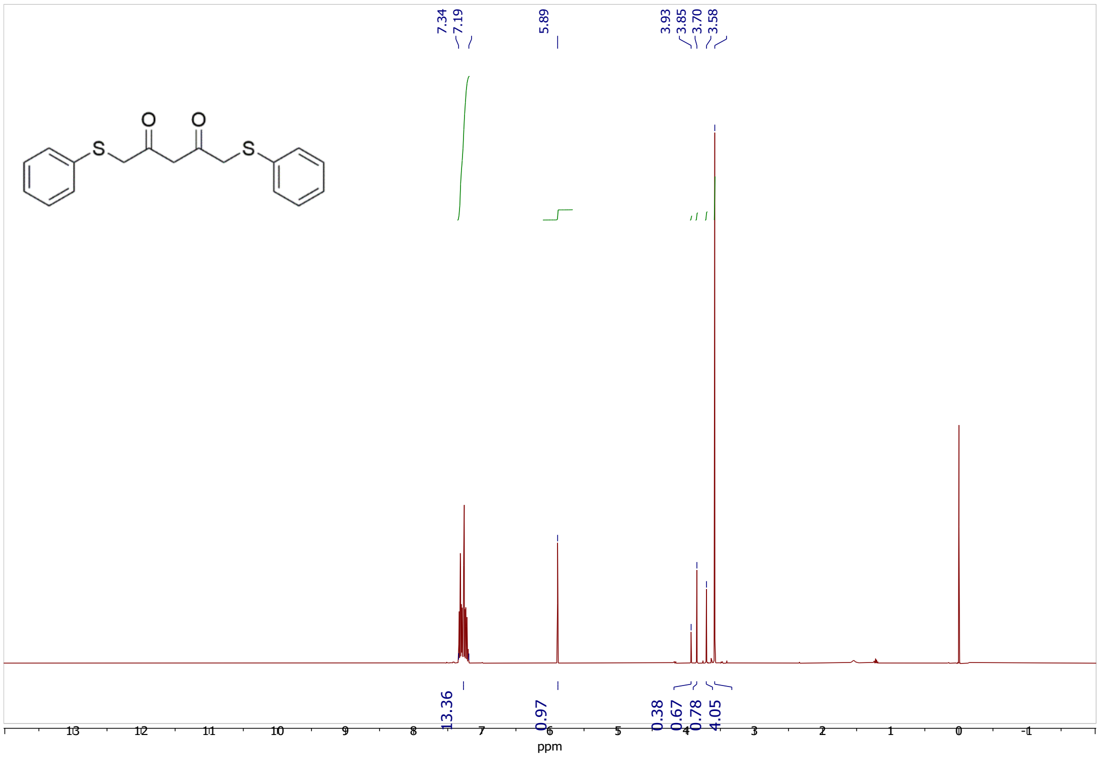

Figure S22: $^{13}C\{^{1}H\}$ NMR of **30** (101 MHz, $CDCl_3$).

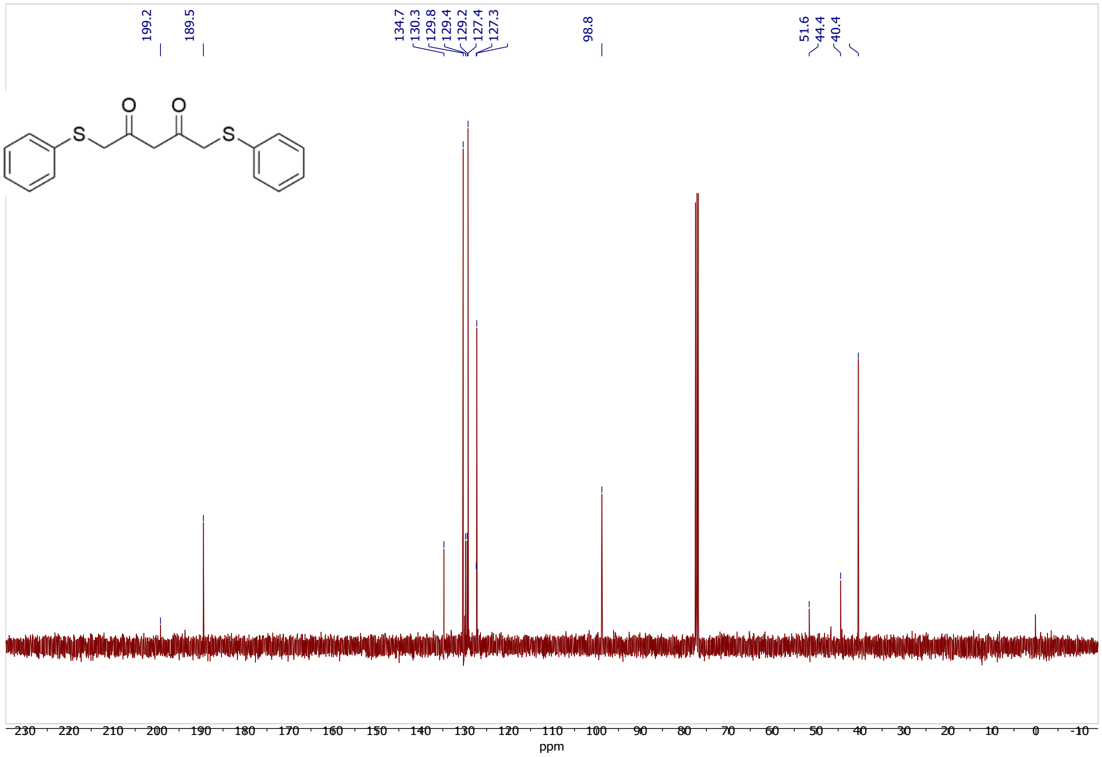

Figure S23: ^{1}H NMR of **32** (400 MHz, $CDCl_3$)

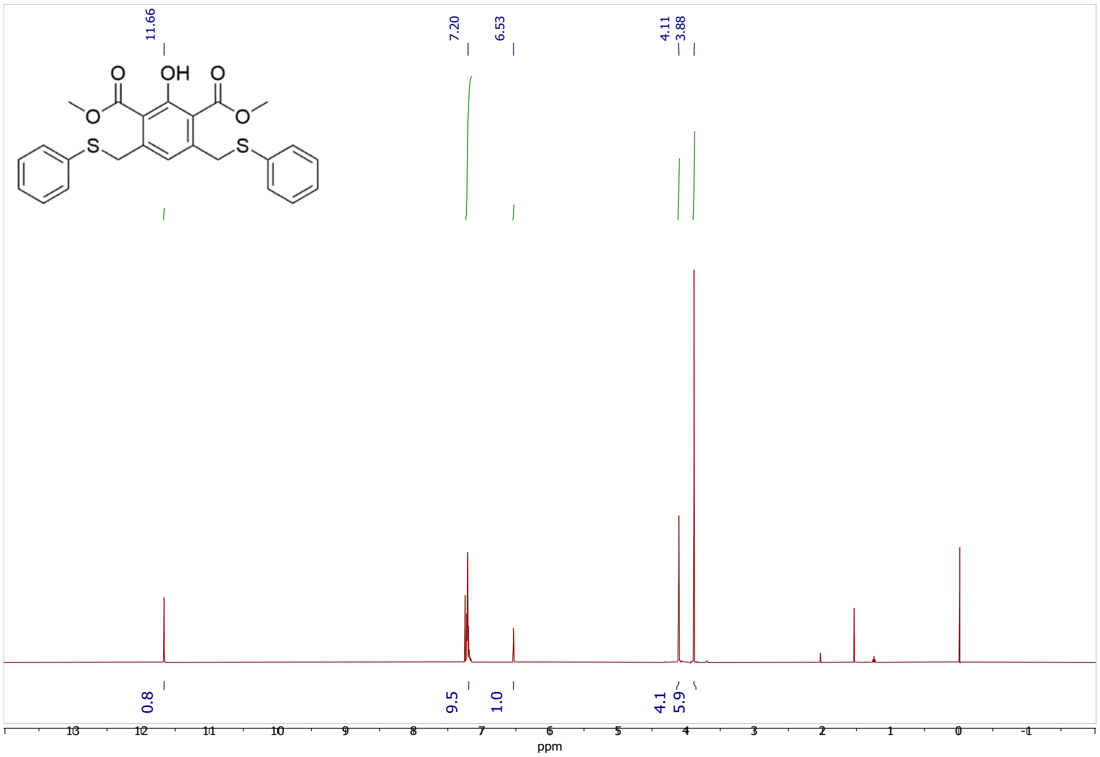

Figure S24: $^{13}C\{^1H\}$ NMR of **32** (101 MHz, $CDCl_3$).

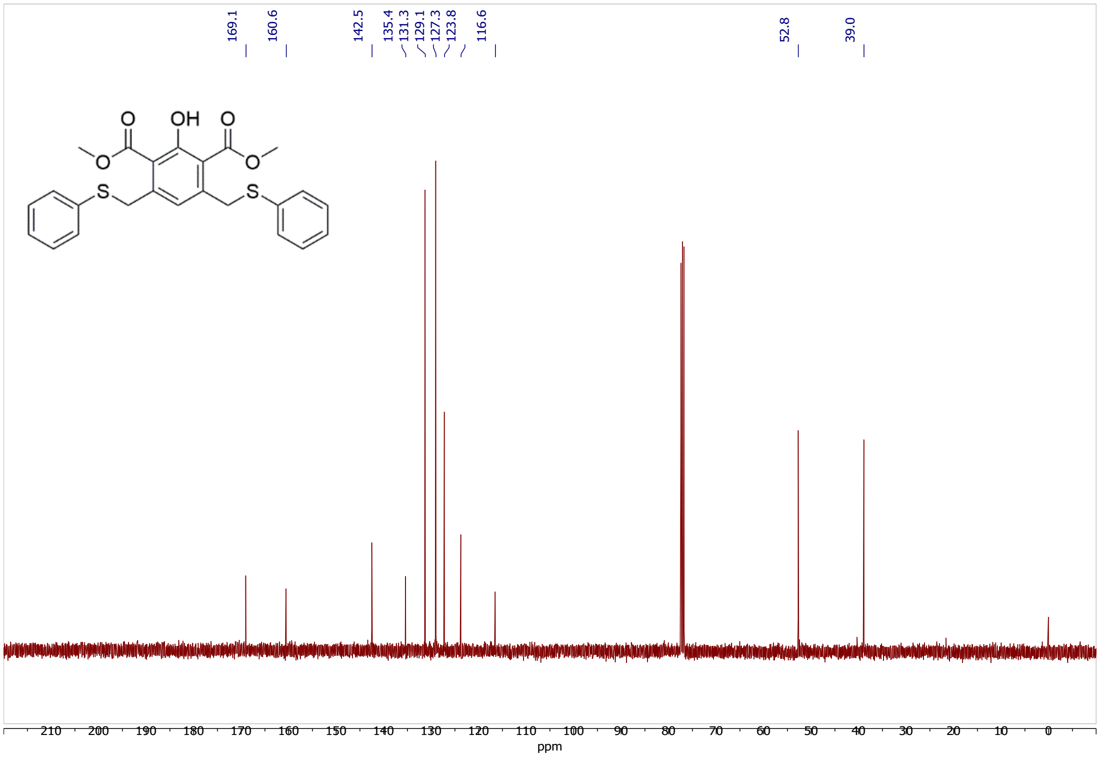

Figure S25: ^{1}H NMR of **33** (400 MHz, $CDCl_3$).

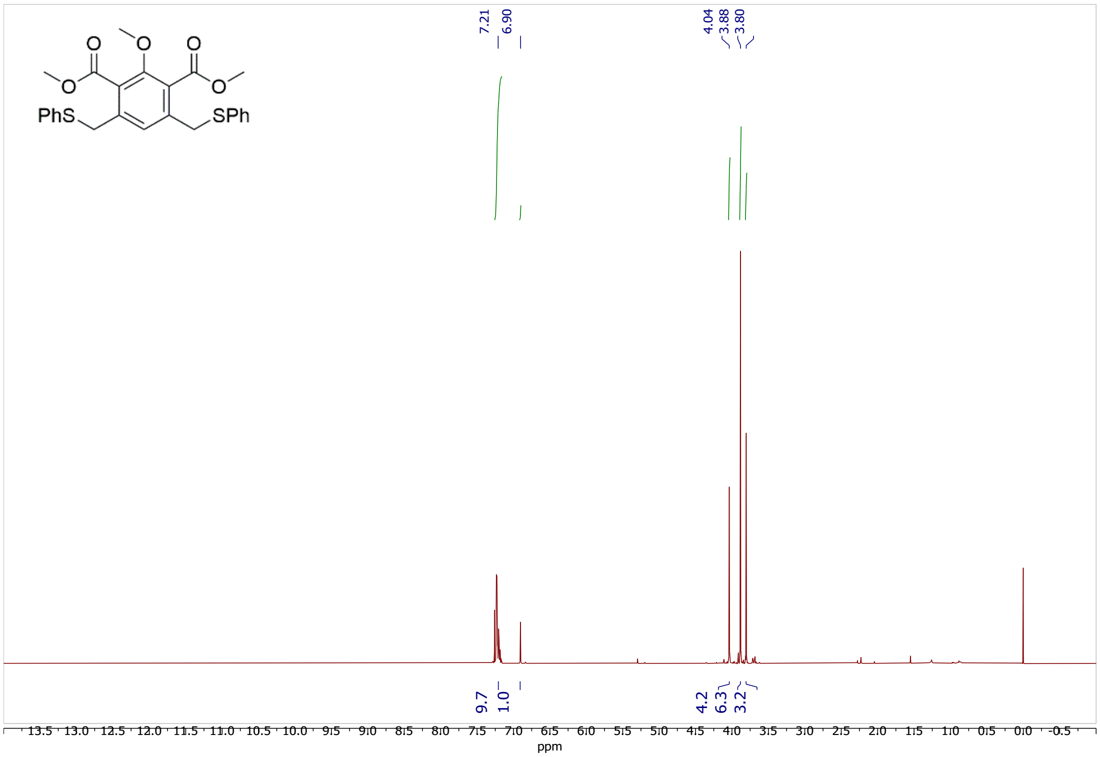

Figure S26: $^{13}C\{^{1}H\}$ NMR of **33** (101 MHz, $CDCl_3$).

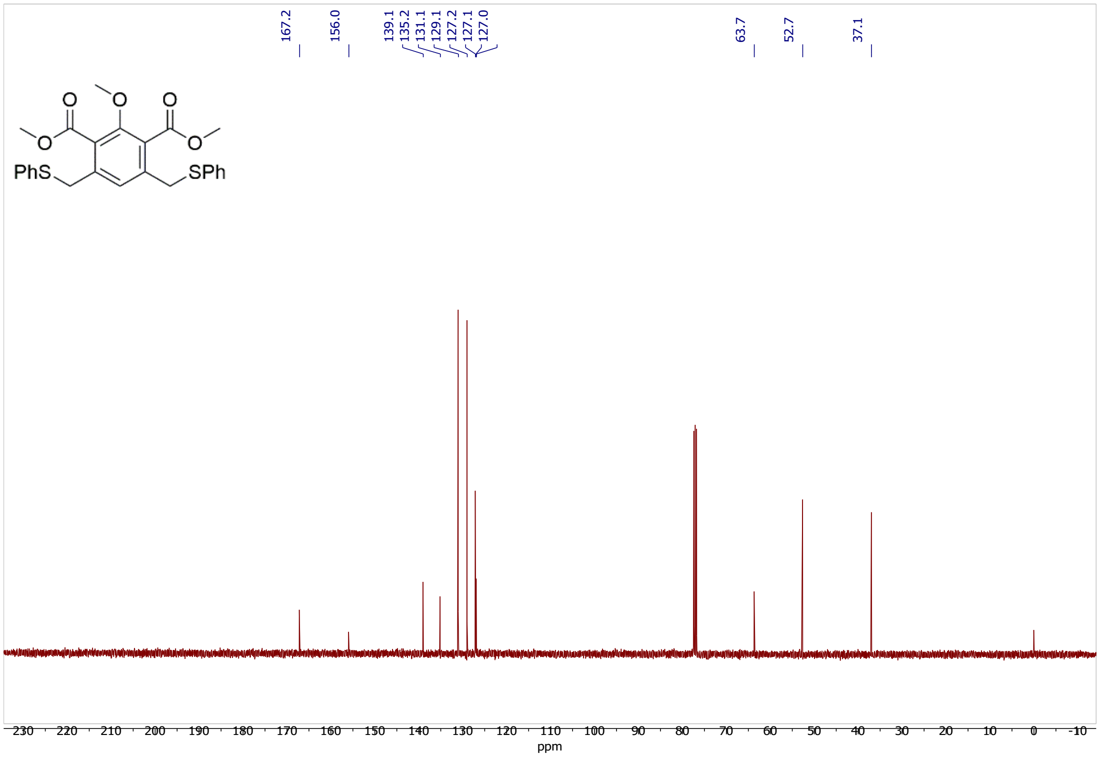

Figure 27: ^{1}H NMR of **18** (400 MHz, $CDCl_3$).

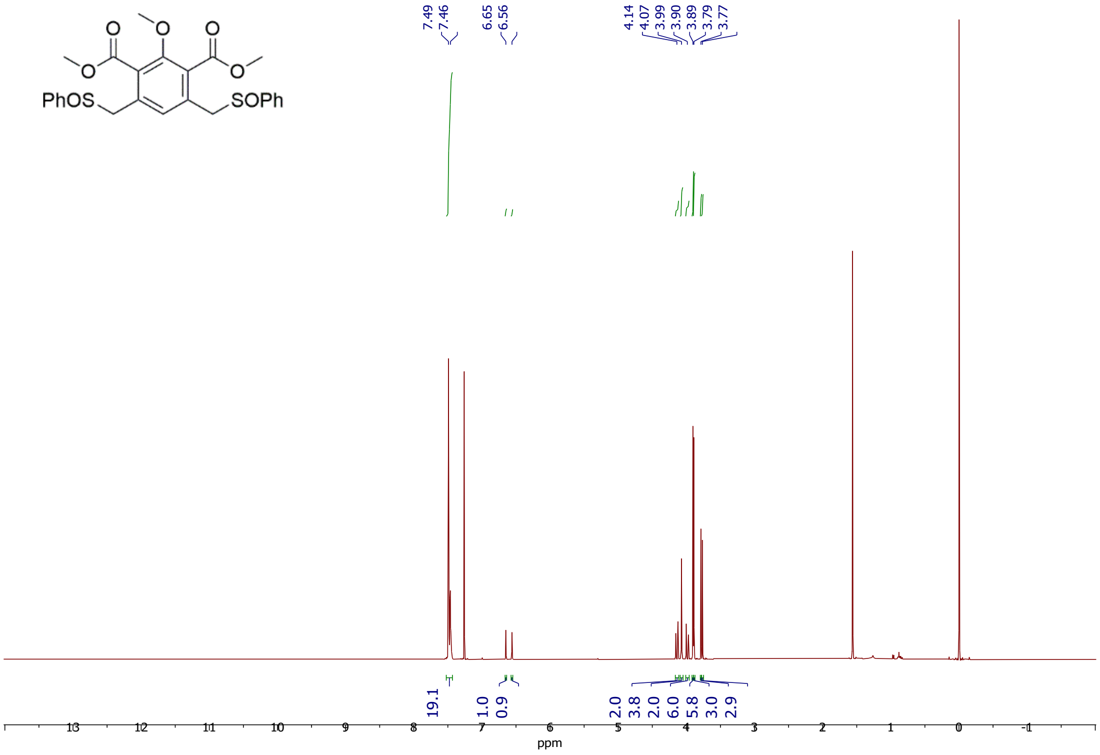

Figure S28: $^{13}C\{^{1}H\}$ NMR of **18** (101 MHz, $CDCl_3$).

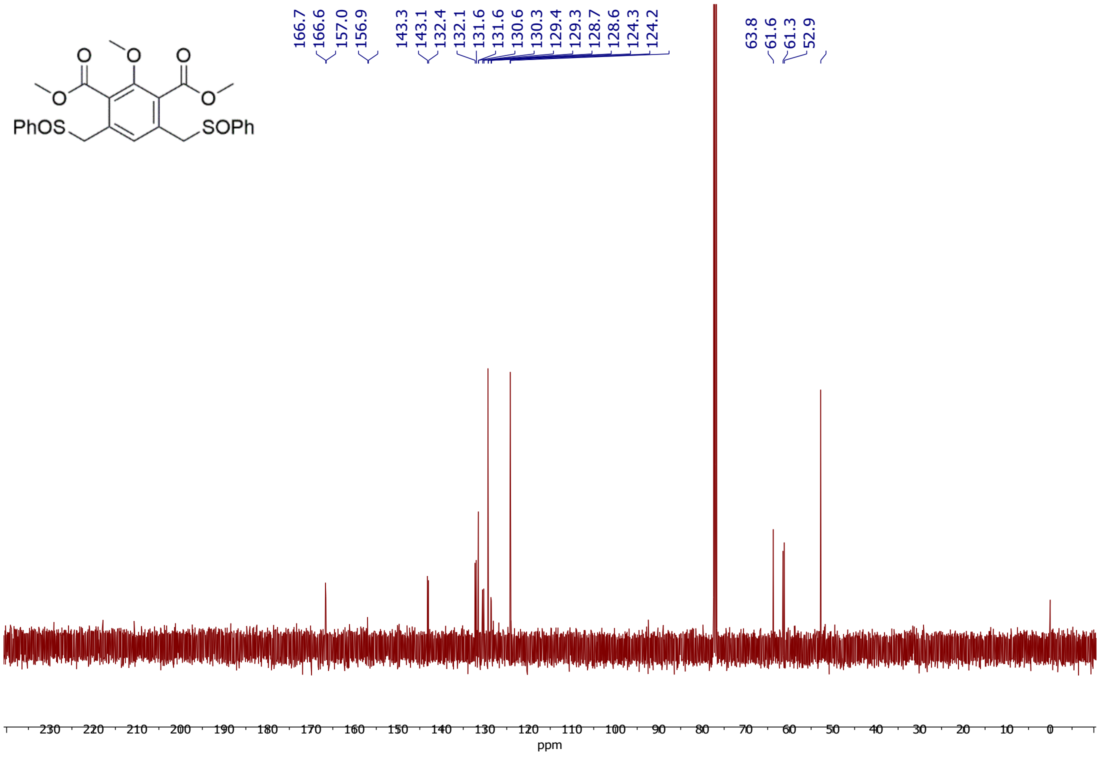

Figure S29: ^{1}H NMR of **34** (400 MHz, $CDCl_3$) plus trace water and grease.

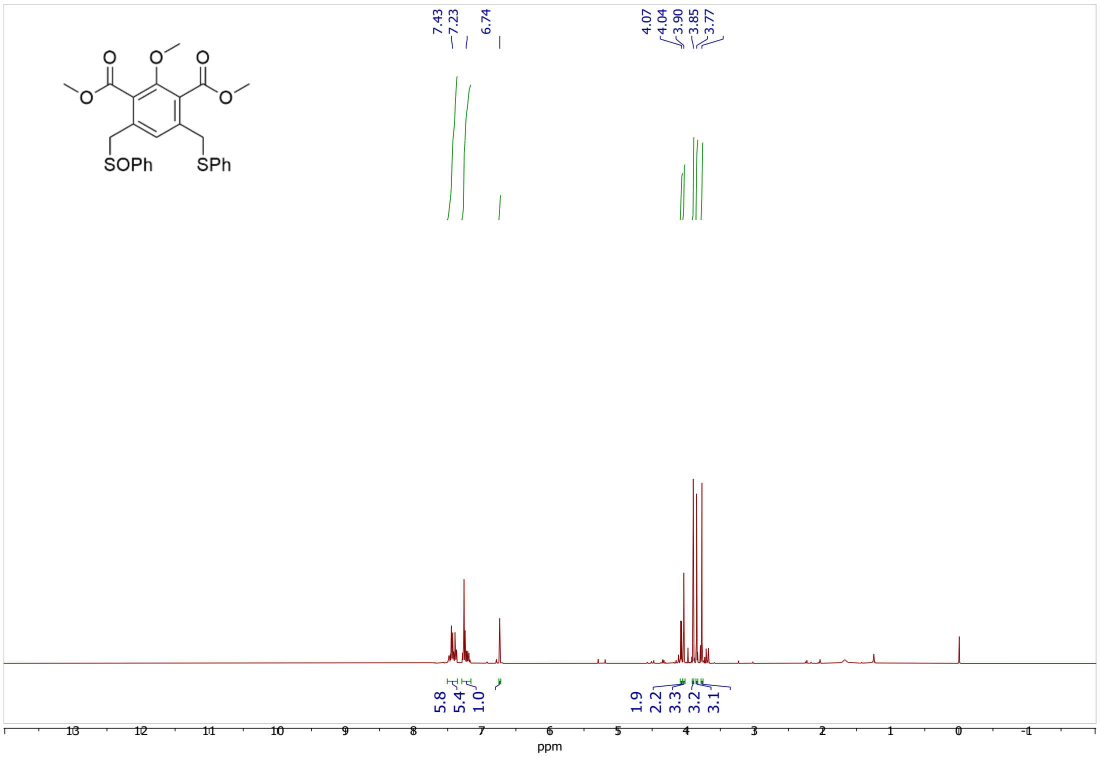

Figure S30: $^{13}C\{^{1}H\}$ NMR of **34** (101 MHz, $CDCl_3$).

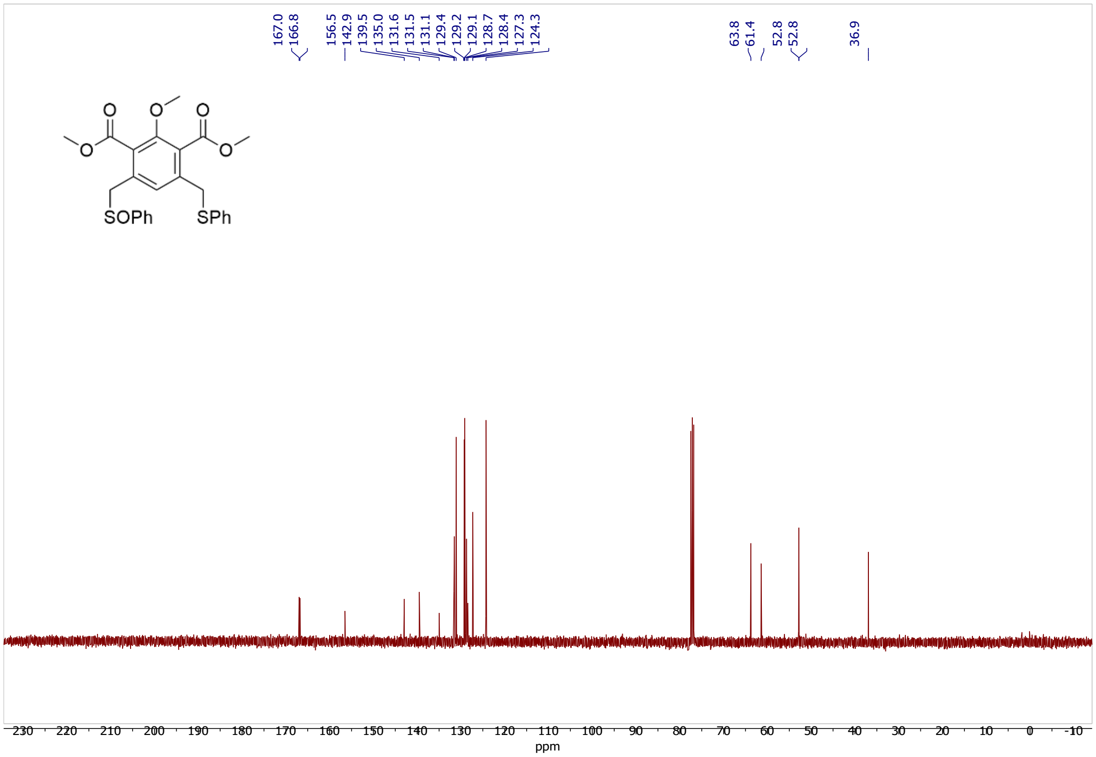

Figure S31: ^{1}H NMR of **20** (400 MHz, $CDCl_3$) plus trace hexane.

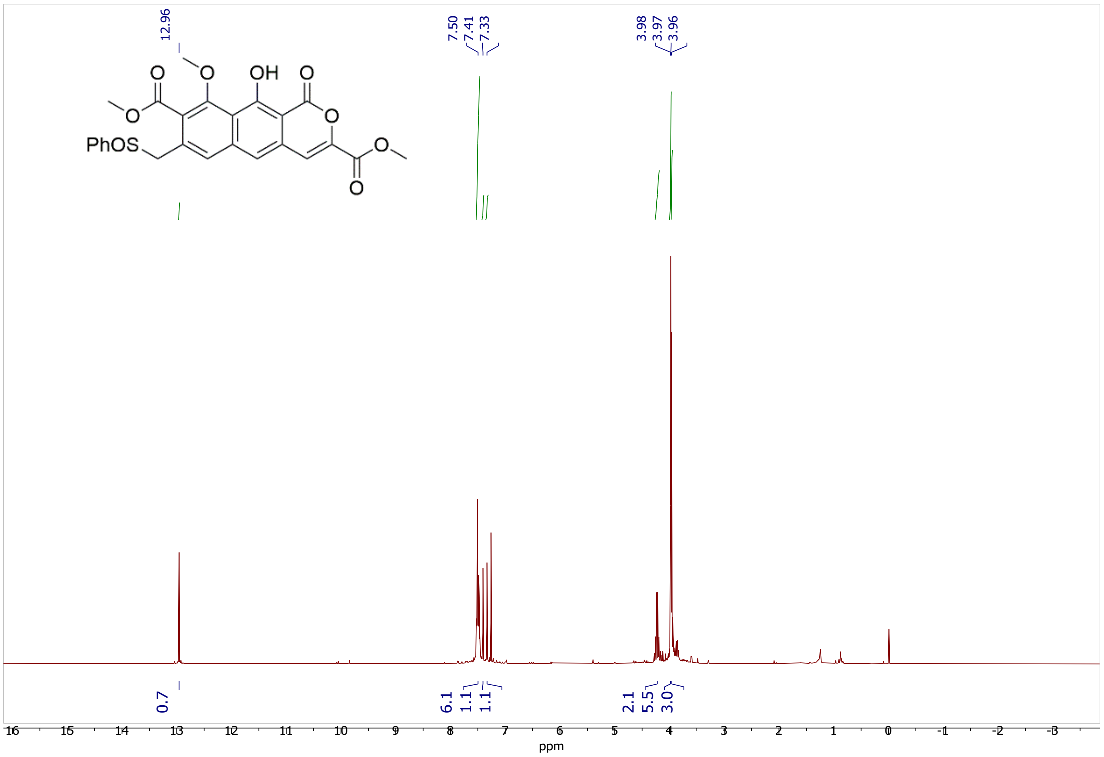

Figure S32: $^{13}C\{^1H\}$ NMR of **20** (101 MHz, $CDCl_3$) plus trace hexane.

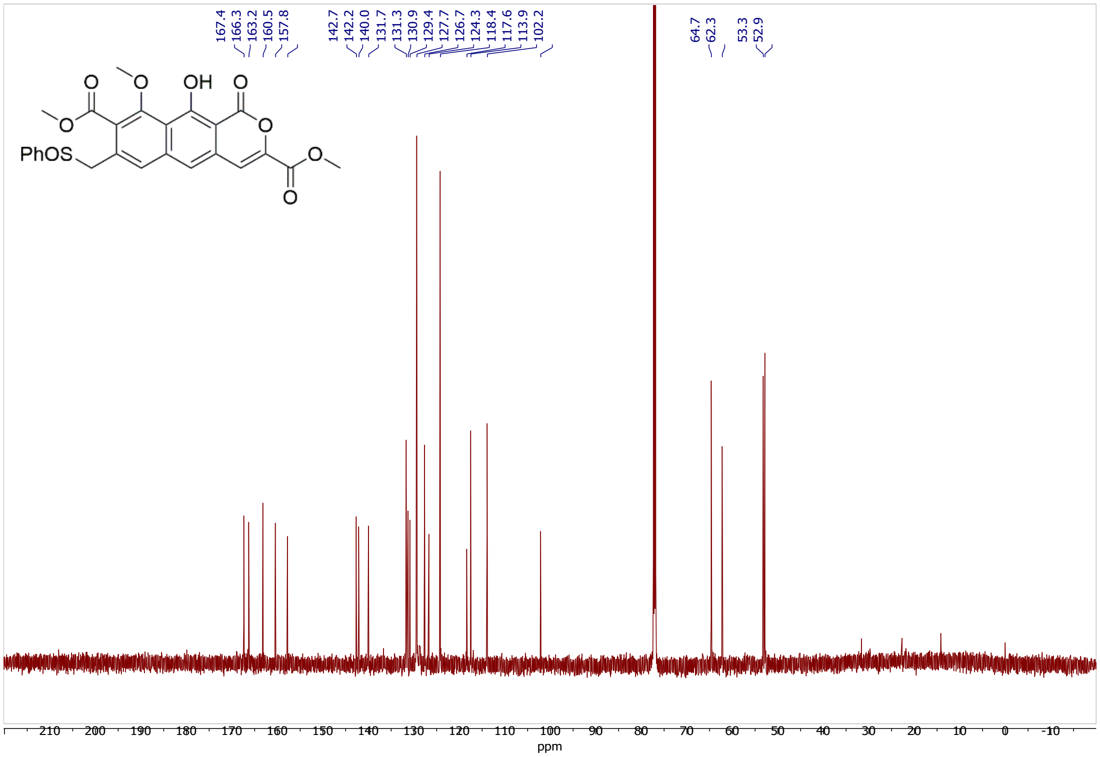

Figure S33: ^{1}H NMR of **35** (100 MHz, $CDCl_3$) plus trace CH_2Cl_2 and EtOAc.

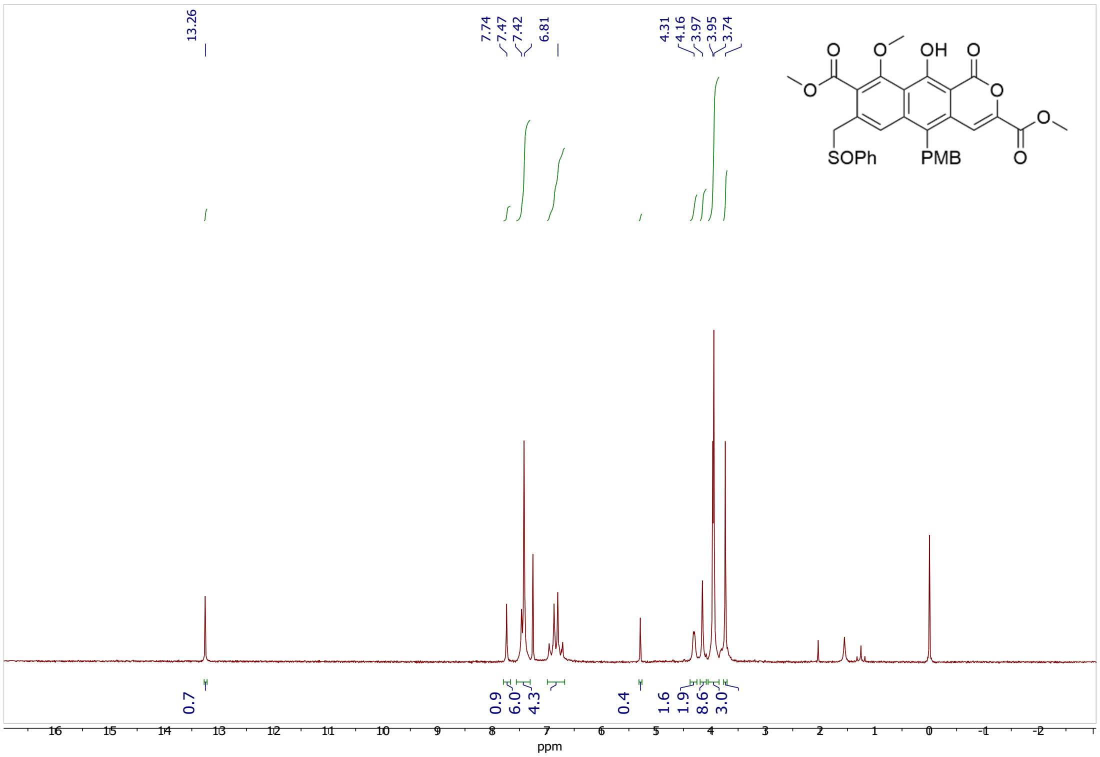

Figure S34: $^{13}C\{^{1}H\}$ NMR of **35** (125 MHz, $CDCl_3$).

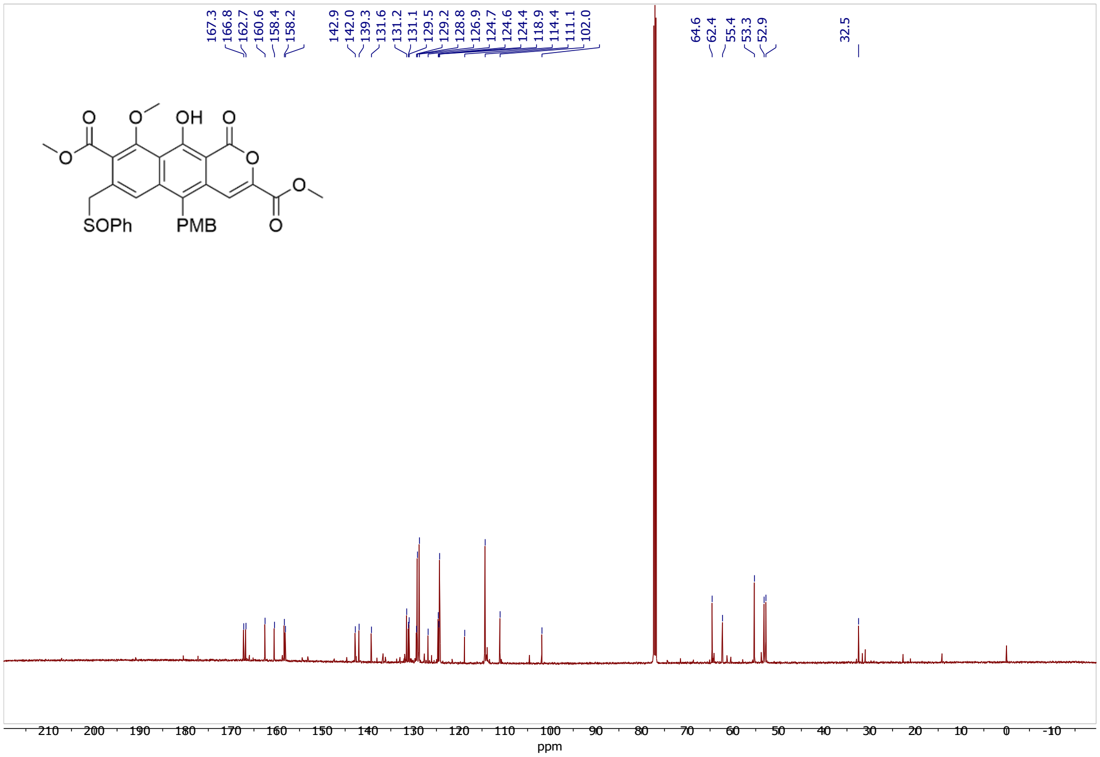

Figure S35: ^{1}H NMR of **36** (400 MHz, $CDCl_3$) plus trace water and grease.

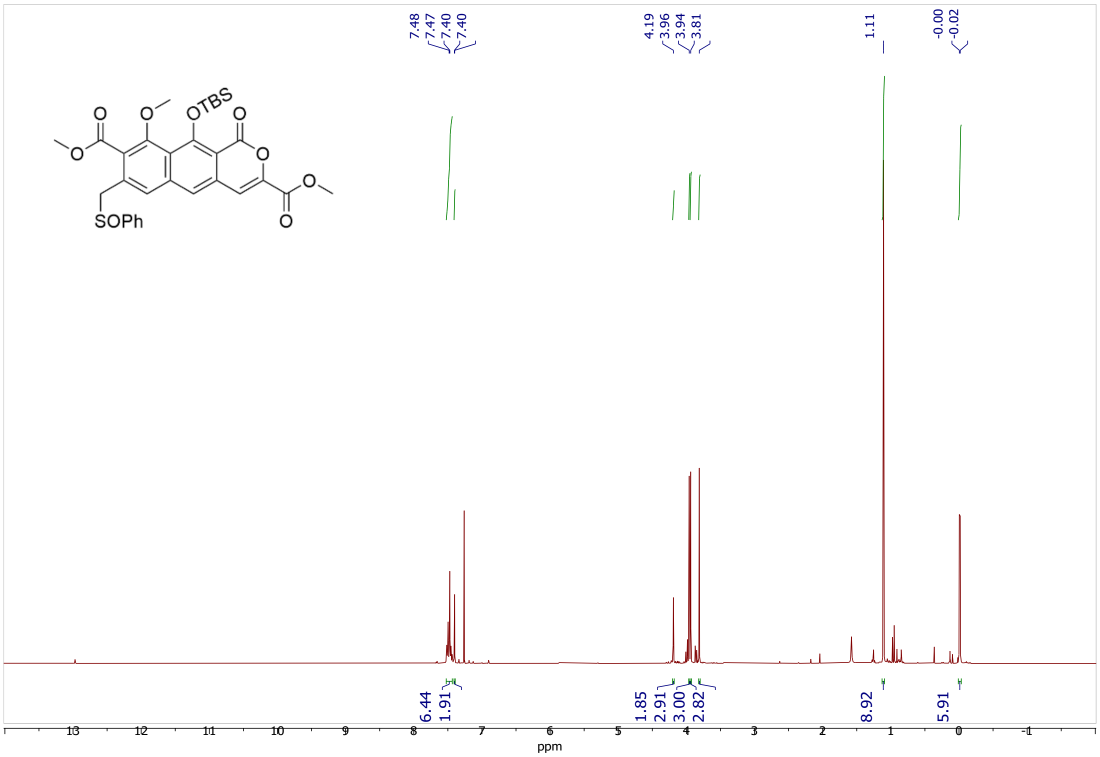

Figure S36: ^{13}C{^{1}H} NMR of **36** (101 MHz, $CDCl_3$).

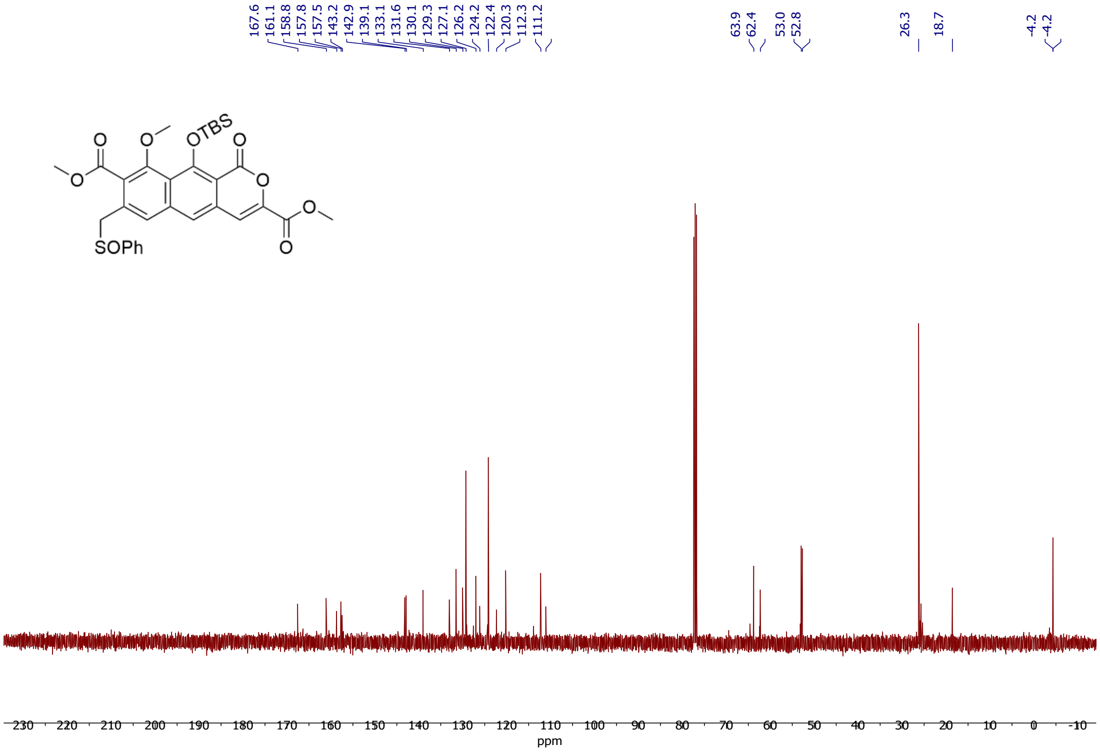

SI References

(1) Evans, G. E.; Leeper, F. J.; Murphy, J. A.; Staunton, J. Triacetic acid lactone as a polyketide synthon: synthesis of toralactone and polyketide-type anthracene derivatives. *J. Chem. Soc., Chem. Commun.* **1979**, 10.1039/c39790000205 (5), 205.

(2) Midland, M. M.; Koops, R. W. Asymmetric hetero Diels-Alder reaction of .alpha.-alkoxy aldehydes with activated dienes. The scope of Lewis acid chelation-controlled cycloadditions. *J. Org. Chem.* **1990**, *55* (17), 5058-5065.

(3) Stetter, H.; Schellhammer, C.-W. Zur Kenntnis Der 2.4-Dioxo-2.3-Dihydro-Pyrane und der 2.4-Dioxo-Tetrahydro-Pyrane. *Justus Liebigs Ann. Chem.* **1957**, *605* (1), 58.

(4) Bertz, S. H. An Improved Synthesis of Some Highly Substituted Phenols - The Prelog Condensation with 2,4,6-Heptanetrione. *Synthesis* **1980**, *1980* (9), 708.

(5) Diemer, V.; Chaumeil, H.; Defoin, A.; Carré, C. Syntheses of extreme sterically hindered 4-methoxyboronic acids. *Tetrahedron* **2010**, *66* (4), 918.

(6) Nwabufo, C. K.; Aigbogun, O. P.; Allen, K. J. H.; Owens, M. N.; Lee, J. S.; Phenix, C. P.; Krol, E. S. Employing in vitro metabolism to guide design of F-labelled PET probes of novel α-synuclein binding bifunctional compounds. *Xenobiotica* **2021**, *51* (8), 885.

(7) Heuser, S. Synthesis of a Highly Functionalised Azepine via a New TBSOTf-Mediated Cyclisation of a Terminal Formamide. *Synlett* **2007**, *2007* (03), 497.

(8) Dickschat, J. S.; Bode, H. B.; Mahmud, T.; Müller, R.; Schulz, S. A novel type of geosmin biosynthesis in myxobacteria. *J. Org. Chem.* **2005**, *70* (13), 5174.

(9) Naghipour, A.; Badpa, K.; Notash, B. From phosphonium salts to binuclear ortho-palladated phosphorus ylides. *Polyhedron* **2015**, *87*, 349.

(10) Dischmann, M.; Frassetto, T.; Breuning, M. A.; Koert, U. Total Synthesis of Isoquinocyclinone. *Chem. Eur. J.* **2014**, *20* (36), 11300.

(11) Wei, H.; Li, Y.; Xiao, K.; Cheng, B.; Wang, H.; Hu, L.; Zhai, H. Synthesis of polysubstituted pyridines via a one-pot metal-free strategy. *Org. Lett.* **2015**, *17* (24), 5974.

(12) Pastine, S. J.; McQuaid, K. M.; Sames, D. Room Temperature Hydroalkylation of Electron-Deficient Olefins: sp3 C−H Functionalization via a Lewis Acid-Catalyzed Intramolecular Redox Event. *J. Am. Chem. Soc.* **2005**, *127* (35), 12180.

(13) Kohanov, Z. A.; Shuvo, S. I.; Lowell, A. N. Regioselective annulation of 6-carboxy-substituted pyrones as a two-carbon unit in formal [4 + 2] cycloaddition reactions. *J. Org. Chem.* **2024**, *89* (13), 9557-9568.

(14) Metanis, N.; Keinan, E.; Dawson, P. E. A designed synthetic analogue of 4-OT is specific for a non-natural substrate. *J. Am. Chem. Soc.* **2005**, *127* (16), 5862.
